# Dictionary Learning Methods for Brain Activity Mapping with MEG Data

**DOI:** 10.1007/s10548-026-01205-7

**Published:** 2026-06-01

**Authors:** Daniela Calvetti, Erkki Somersalo

**Affiliations:** Case Western Reserve University, Department of Mathematics, Applied Mathematics, and Statistics, Cleveland, OH USA

## Abstract

A central goal in many brain studies is the identification of those brain regions that are activated during an observation window that may correspond to a motor task, a stimulus, or simply a resting state. While functional MRI is currently the most commonly employed modality for such task, methods based on the electromagnetic activity of the brain are valuable alternatives because of their excellent time resolution and of the fact that the measured signals are directly related to brain activation and not to a secondary effect such as the hemodynamic response. In this work we focus on the MEG modality, investigating the performance of a recently proposed Bayesian dictionary learning (BDL) algorithm for brain region identification. The partitioning of the source space into the 148 regions of interest (ROI) corresponding to parcellation of the Destrieux atlas provides a natural determination of the subdictionaries necessary for the BDL algorithm. We design a simulation protocol where a small randomly selected patch in each ROI is activated, the MEG signal is computed and the inverse problem of active brain region identification is solved using the BDL algorithm. The BDL algorithm consists of two phases, the first one comprising dictionary compression and Bayesian compression error analysis, and the second one performing dictionary coding with a deflated dictionary built on the output of the first phase, both steps relying on Bayesian sparsity promoting computations. For assessing the performance, we give a probabilistic interpretation of the confusion matrix, and consider different impurity measures for a multi-class classifier.

Key words: Sparse coding, Bayesian approximation error, Matrix factorization, Impurity index, Bayesian hierarchical models

## Introduction

Understanding the connectivity and the functional organization of the human brain is an important aspect of brain research, including studies on consciousness, sleep, autism, a range of neuro-degenerative diseases, language development, intelligence and more. Understanding the network structure of the brain may also help understanding how epileptic seizures are triggered. An essential part of this endeavor is parcellation of the brain into regions that can be identified based on either brain anatomy or brain function. While the overall functional role of some brain areas, for example motor cortex, olfactory bulb, or visual cortex, is well understood, a finer parcellation and the association of detected activation with a specific function is challenging because of the lack of definite ground truth, possibly leaving room for different interpretations. In summary, although automatic anatomy-based parcellation techniques have been proposed in the literature, see, e.g., Lancaster et al. (2000); Desikan et al. (2006); Destrieux et al. (2010), unambiguous identification of functional areas is more challenging, as pointed out, e.g., in Salehi et al. (2020). In particular, the functional organization of the brain may be hierarchical and multi-scale in nature as some studies indicate (Doucet et al. 2011; Hilgetag and Goulas 2020).

Most functional brain mapping studies are based on PET or fMRI imaging, see, e.g. Raichle (2009) for a brief history of functional brain mapping. Currently, fMRI is the main imaging tool in cognitive neuroscience, however, its limitations are known and have been discussed in the literature (Logothetis 2008). In particular, it is well understood that the fMRI modality is sensitive to the oxygenation level of the blood, which does not directly correspond to cerebral metabolic rate (CMR) of oxygen, raising the question of its direct correspondence to brain activity. The interpretation of the BOLD data as a direct proxy for neuronal firing activity is further complicated by the fact that inhibitory activity increases CMR but is not associated with neuronal firing, implying that fMRI may be unable to distinguish excitation and inhibition unequivocally. Magnetoencephalography (MEG), on the other hand, seeks to estimate directly the neuronal electrophysiological synaptic activity, thereby offering a viable alternative for cognitive neuroimaging. One question that needs to be addressed with MEG is whether it can correctly identify active brain regions, which is the main topic of this study.

Unlike in the standard MEG inverse problem of source localization (Baillet et al. 2001; Hämäläinen et al. 1993), we consider the problem of identification of active brain regions based on MEG data, i.e., singling out a few brain regions containing the electric currents inducing the magnetic field within the observation accuracy. In particular, different sources in the same brain region are considered equivalent, and the goal is to identify as few as possible regions so that the data are satisfactorily explained. The proposed algorithm therefore involves sparse coding in terms of the brain regions specified by a given brain atlas. In the context of MEG and EEG, sparsity-promoting methods have been considered for decades, either based on appropriately chosen penalties, see, e.g., Uutela et al. (1999); Krishnaswamy et al. (2017); Pascarella et al. (2019), or more in line with the current analysis, using Bayesian sparsity-promoting priors (Calvetti et al. 2015, 2019, 2023). In the current setting, rather than looking for a sparse representation of the activated source in terms of single dipoles, we seek to explain the data in terms of a few groups of sources, yielding a group sparsity problem. Group sparsity-promoting penalty methods have been previously proposed in the context of MEG, see, e.g., Lim et al. (2017). In this work, the group sparsity methods are developed further in the Bayesian framework, and in particular, in the dictionary-based setting, where the dictionary interpretation is guided by an underlying brain parcellation. One way to lessen the computational burden is to first perform a compression of the dictionary, which can be regarded as a model reduction technique and, following the ideas of Bayesian approximation error analysis (Kaipio and Somersalo 2006, 2007), augment the compressed dictionary with the associated compression error. A statistically motivated group sparsity prior, similar to the anatomical prior proposed in Calvetti et al. (2015), is introduced to improve the success rate of the algorithm. Following (Bocchinfuso et al. 2024), the compressed dictionary matching algorithm is used to extract from the original dictionary only the portion that may be pertinent to explain the MEG data. This leads to a two-phase algorithm based on recent ideas of Bayesian sparsity promotion. The proposed algorithm is utilized in a large set of numerical simulations designed to test how well MEG can be used to detect activation in the different brain regions.

In this article, we adopt the following notional convention. Random variables are denoted by upper case letters, and their realizations by the corresponding lower case letters. To distinguish from scalar quantities, physical vectors such as dipoles, and Euclidean (column) vectors are denoted by boldface letters. we reserve the sans serif fonts (e.g., ${\mathsf A}$, ${\mathsf B}$) for matrices, while $\Vert \,\cdot \,\Vert$ denotes the standard Euclidean norm of a vector.

## Brain Activity Mapping

A standard model for the electromagnetic brain activity represents the impressed current associated with the post-synaptic ion currents in the brain as a discrete sum of point-like dipoles,

1 $$\begin{aligned} {\boldsymbol{J}}= \sum _{k=1}^N {\boldsymbol{q}}_k, \end{aligned}$$

where ${\boldsymbol{q}}_k\in {\mathbb {R}}^3$ is the moment of the *k*th dipole located at ${\boldsymbol{p}}_k\in {\mathbb {R}}^3$. The ensemble of dipole positions constitutes the source space $S = \{{\boldsymbol{p}}_k:1\le k\le N\}$. In the present article we consider the magnetoencephalography (MEG) data, comprising the noisy observation of the magnetic flux density components by a number of magnetometers, augmented by the gradiometer data, i.e., gradients of the flux densities in the direction perpendicular to the magnetometer axis. Let *m* denote the number of data channels. The quasi-static Maxwell’s equations allow us to represent the measured components of the magnetic flux density and their tangential gradients as

2 $$\begin{aligned} {{\boldsymbol{b}}} = \sum _{k=1}^N {\mathsf L}_k {{\boldsymbol{q}}}_k, \end{aligned}$$

where$\textbf{q}_k$ is the column vector with the Cartesian components of the *k*th dipole as its entries, ${{\boldsymbol{b}}}\in {\mathbb {R}}^m$ is the vector of all observed scalar quantities, and ${\mathsf L}_k\in {\mathbb {R}}^{m\times 3}$ is the lead field matrix accounting for the geometry and conductivity distribution of the head; for details about modeling and computations, see, e.g., Ilmoniemi and Sarvas (2019); Wolters et al. (2004).

While the exact location of the brain activity is crucial in applications such as planning epilepsy surgery, to understand the time course of brain functions during a task or in the resting state, or to map the functional connectivity of the brain, it is often sufficient to identify the active brain region within a given brain atlas. In that context, it is natural to approach the problem from the point of view of dictionary learning and dictionary matching.

### Dictionary Matching with Group Sparsity

For simplicity, consider the forward model (2) under the simplifying hypothesis that the dipole directions are fixed to the preferential direction, typically normal to the cortex,; see, e.g. Giri et al. (2025). Denoting by $\widehat{{\boldsymbol{q}}}_k\in {\mathbb {S}}^2$ the unit vector determining the preferential direction at the source space location ${\boldsymbol{p}}_k$, we write the forward model as

3 $$\begin{aligned} {{\boldsymbol{b}}} = \sum _{k=1}^N \big (\pm \Vert {{\boldsymbol{q}}}_k\Vert \big ) \big ({\mathsf L}_k \widehat{{\boldsymbol{q}}}_k\big ). \end{aligned}$$

ignoring, for now, the effects of observation noise and unspecified modeling uncertainties that are not included in the representation. Furthermore, we assume that the dipole and signal strengths are of secondary interest. Therefore, given an observation vector ${\boldsymbol{b}}$, we consider the *dictionary matching problem* of finding a representation

4 $$\begin{aligned} {{\boldsymbol{y}}} = \sum _{k=1}^N {{\boldsymbol{d}}}_k x_k = {\mathsf D}{{\boldsymbol{x}}}, \end{aligned}$$

where

5 $$\begin{aligned} {{\boldsymbol{y}}} = \frac{{\boldsymbol{b}}}{\Vert {{\boldsymbol{b}}}\Vert }, \quad {{\boldsymbol{d}}}_k = \frac{{\mathsf L}_k \widehat{{\boldsymbol{q}}}_k}{\Vert {\mathsf L}_k \widehat{{\boldsymbol{q}}}_k\Vert }, \quad {\mathsf D}= \left[ \begin{array}{ccc} {{\boldsymbol{d}}}_1&\cdots&{{\boldsymbol{d}}}_N\end{array}\right] , \quad {{\boldsymbol{x}}} = \left[ \begin{array}{c} x_1 \\ \vdots \\ x_N\end{array}\right] \end{aligned}$$

ensuring that the columns of the matrix ${\mathsf D}\in {\mathbb {R}}^{m\times N}$ are scaled to have unit Euclidean norm and the coefficients $x_k$ are proportional to the amplitudes of the corresponding dipoles. The recasting (4) of the source identification problem amounts to matching the query ${{\boldsymbol{y}}}$, the scaled MEG data, with the dictionary atoms ${{\boldsymbol{d}}}_k$ encoding the dipole contributions.

A physiological atlas of the brain comprising *L* regions, given in terms of disjoint partitioning of the index set

6 $$\begin{aligned} I =\big \{1,2,\ldots ,N\big \} = \bigcup _{\ell =1}^L I_\ell , \quad I_\ell \cap I_{\ell '} = \emptyset \text{ for } \ell \ne \ell '\text{, } \end{aligned}$$

can be used to obtain a natural clustering of the dictionary atoms so that we can reformulate the dictionary matching problem as

7 $$\begin{aligned} {{\boldsymbol{y}}} = \sum _{\ell =1}^L\left( \sum _{k\in I_\ell } {{\boldsymbol{d}}}_k x_k\right) =\sum _{\ell = 1}^L {\mathsf D}^{(\ell )} {{\boldsymbol{x}}}^{(\ell )}. \end{aligned}$$

Here, ${\mathsf D}^{(\ell )}$ is a matrix with columns ${\boldsymbol{d}}_k$ with $k\in I_\ell$. If the number of indices in $I_\ell$ is $n_\ell$, we have ${\mathsf D}^{(\ell )}\in {\mathbb {R}}^{m\times n_\ell }$. The matrix ${\mathsf D}^{(\ell )}$ is called the *ℓ*th subdictionary, its columns ${\boldsymbol{d}}_k$ with $k\in I_\ell$ are the atoms of the subdictionary, and ${\boldsymbol{x}}^{(\ell )}\in {\mathbb {R}}^{n_\ell }$ is the vector containing the corresponding weights. If the brain activity at a given time instance is believed to be restricted into only few brain regions, it is reasonable to seek a solution such that the vector $\big (\Vert {{\boldsymbol{x}}}^{(1)}\Vert , \ldots , \Vert {{\boldsymbol{x}}}^{(L)}\Vert \big )$ is sparse, i.e., most of the coefficient vectors ${{\boldsymbol{x}}}^{(\ell )}$ are zero or at least negligible. This is tantamount to a *group sparsity* prior belief. In the literature, group sparsity has often been promoted through group LASSO regularization (Yuan and Lin 2006), leading to the minimization problem

8 $$\begin{aligned} \min _{{\boldsymbol{x}}} \Vert {{\boldsymbol{y}}} - {\mathsf D}{{\boldsymbol{x}}}\Vert ^2 + \lambda \sum \Vert {{\boldsymbol{x}}}^{(\ell )}\Vert , \end{aligned}$$

where *λ>0* is a properly chosen Lagrange multiplier that can be interpreted as a Tikhonov type regularization parameter. Our approach to the promotion of group sparsity is through computationally efficient hierarchical models. More specifically, we propose a two-phase classifier algorithm based on a Bayesian model for dictionary compression and deflation. In the following section, we outline the structure of the algorithm. The details of its implementations will be presented in Sect. 3.

### A Two-Phase Classifier: An Outline of the Algorithm

We begin by assuming that the dictionary matrix ${\mathsf D}$ defined in (5) has been partitioned into subdictionaries ${\mathsf D}^{(\ell )}$,

9 $$\begin{aligned} {\mathsf D}= \left[ \begin{array}{ccc} {\mathsf D}^{(1)}&\cdots&{\mathsf D}^{(L)} \end{array}\right] \end{aligned}$$

each subdictionary corresponding to a region of an underlying brain atlas.

**Dictionary compression:** In dictionary matching and learning algorithms it is common to compress the subdictionaries to summarize their content in terms of a few feature vectors, thus reducing the degrees of freedom. Dictionary compression often is based on a low-rank approximation of each subdictionary,

10 $$\begin{aligned} {\mathsf D}^{(\ell )} \approx {\mathsf W}^{(\ell )} {\mathsf H}^{(\ell )}, \; {\mathsf W}^{(\ell )} \in {\mathbb {R}}^{m \times r_\ell }, \; {\mathsf H}^{(\ell )} \in {\mathbb {R}}^{r_\ell \times n_\ell } \end{aligned}$$

using, e.g., a truncated singular value decomposition (SVD) or non-negative matrix factorization (NMF) (Aharon et al. 2006; Gillis 2020). The *ℓ*th compressed dictionary ${\mathsf W}^{(\ell )}$, also called the code book, comprises of few feature vector columns that approximately span the range of the full subdictionary ${\mathsf D}^{(\ell )}$. The number $r_\ell$ of the feature vectors included in the compressed dictionary depends on the class, and it is chosen so that the compressed dictionary is representative enough to approximate the range of the subdictionary.

**Phase I: Identification of relevant subdictionaries:** In the first phase of the algorithm, the goal of the sparse compressed dictionary learning is to find a representation

11 $$\begin{aligned} {{\boldsymbol{y}}} \approx \sum _{\ell = 1}^L {\mathsf W}^{(\ell )} {{\boldsymbol{z}}}^{(\ell )} \end{aligned}$$

such that most of the coefficient vectors ${{\boldsymbol{z}}}^{(\ell )}\in {\mathbb {R}}^{r_\ell }$ are zero or negligible in size, that is, the sum (11) reduces to

12 $$\begin{aligned} \textbf{y} \approx \sum _{j = 1}^q {\mathsf W}^{(\ell _j)} \textbf{z}^{(\ell _j)}, \quad q\ll L, \end{aligned}$$

for few dictionary indices $\ell _1,\ldots, \ell _q$. Effectively, this phase identifies a minimal subset of subdictionaries that are necessary to explain the data.

**Dictionary deflation**: Having identified the necessary subdictionaries with indices $\ell _1, \ldots ,\ell _q$, we define a reduced, or deflated dictionary,

13 $$\begin{aligned} {\mathsf D}^\textrm{defl} = \left[ \begin{array}{ccc} {\mathsf D}^{(\ell _1)}&\cdots&{\mathsf D}^{(\ell _q)}\end{array}\right] , \end{aligned}$$

comprising only the subdictionaries deemed as pertinent.

**Phase II: Best explaining dipole identification:** Having discarded the irrelevant subdictionaries, in the second phase we seek to find an optimal representation of the data vector in terms of a few atoms of the reduced dictionary,

14 $$\begin{aligned} {{\boldsymbol{y}}} \approx {\mathsf D}^\textrm{defl} {{\boldsymbol{x}}}, \quad {{\boldsymbol{x}}} \text{ sparse, } \end{aligned}$$

which is formally identical to source localization with a reduced lead field. For the identification of the active brain region, we apply the winner-takes-all classification strategy, associating the data ${{\boldsymbol{y}}}$ with the subdictionary that contains the best matching dipole source.

**Fig. 1:**
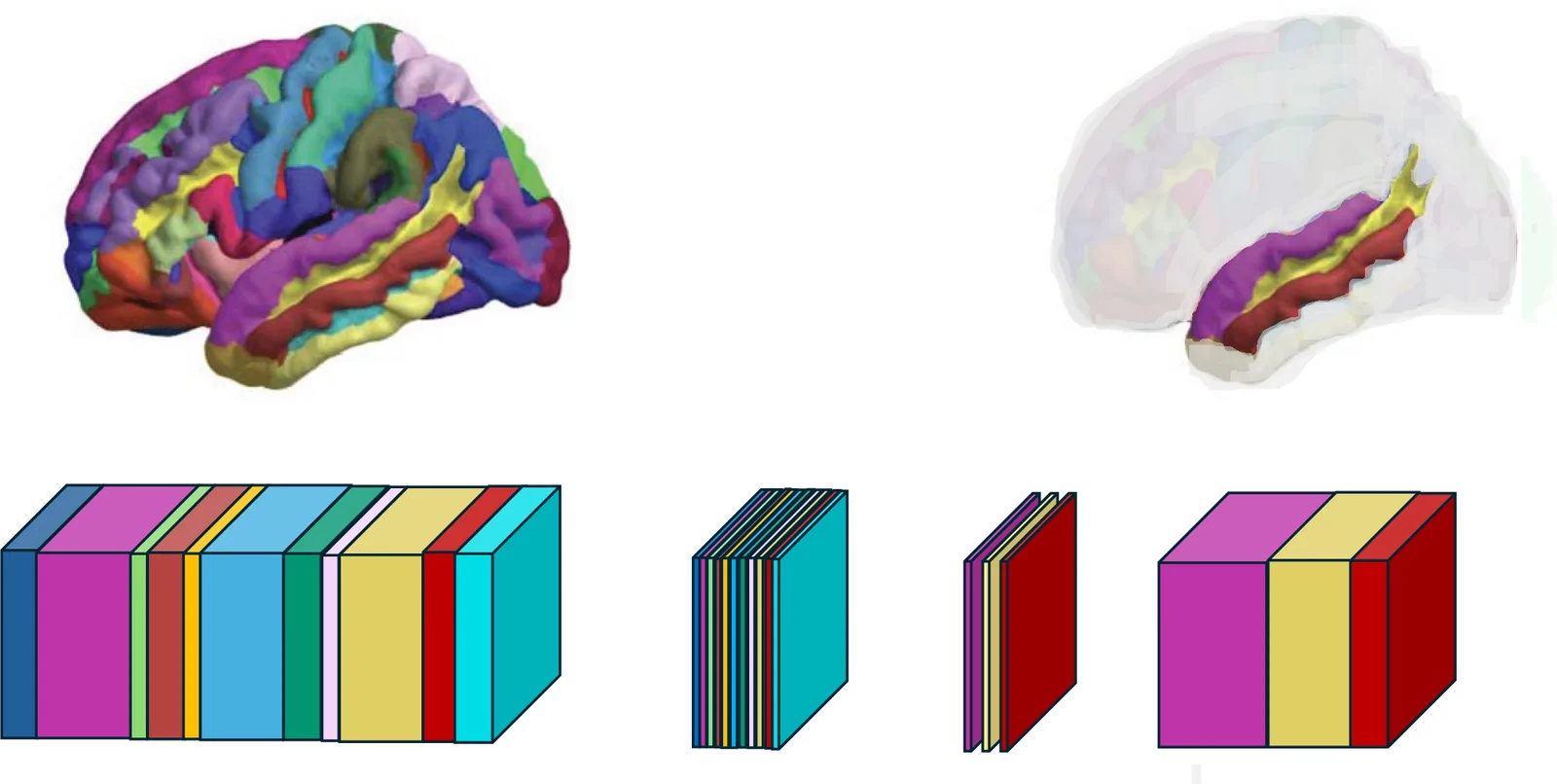
Schematic representation of the algorithm. The brain atlas (top left) gives rise to a decomposition of the dictionary into subdictionaries ${\mathsf D}^{(\ell )}$ that are schematically represented as color coded boxes on bottom left. The dictionary compression reduces each subdictionary into a thin code book ${\mathsf W}^{(\ell )}$. Phase I of the algorithm selects a sparse set of relevant code books corresponding to only few brain regions, as indicated on top right. Phase II identifies the most relevant brain region based on dictionary matching of the data with the deflated dictionary, symbolically shown on bottom right

The proposed algorithm is schematically summarized in Fig. 1.

In the following section, we present discuss the algorithmic details of each step.

## Algorithmic Details

This section gives the details of the implementation of the two-phase algorithm that follows the lines of the classifier in Bocchinfuso et al. (2024). The algorithm is based on the Bayesian paradigm of inverse problems, in which unknowns are modeled as random variables, the randomness reflecting the uncertainty about their values, and encoded in the probability distributions.

### Dictionary Compression

We review here a dictionary compression technique leveraging ideas from the Bayesian inverse problems paradigm. We start by writing the singular value decomposition (SVD) of each subdictionary matrix,

15 $$\begin{aligned} {\mathsf D}^{(\ell )} = {\mathsf U}^{(\ell )} {\mathsf \Sigma }^{(\ell )} \left( {\mathsf V}^{(\ell )}\right) ^{\mathsf T}, \end{aligned}$$

where ${\mathsf U}^{(\ell )} \in {\mathbb {R}}^{m\times m}$ and ${\mathsf V}^{(\ell )}\in {\mathbb {R}}^{n_\ell \times n_\ell }$ are orthogonal matrices and ${\mathsf \Sigma }\in {\mathbb {R}}^{m\times n_\ell }$ is diagonal with non-negative diagonal entries $\sigma _j^{(\ell )}$ appearing in decreasing order. For each *ℓ*, we write a low-rank approximation of the subdictionary by discarding singular triplets associated with singular values values below a given percentage *τ*, *0<τ <1*, of the largest one,

16 $$\begin{aligned} {\mathsf D}^{(\ell )} \approx \sum _{j=1}^{r_\ell } \sigma _j^{(\ell )} {\boldsymbol{u}}^{(\ell )}_j \big ({\boldsymbol{v}}^{(\ell )}_j\big )^{\mathsf T}= {\mathsf W}^{(\ell )} {\mathsf H}^{(\ell )},\quad \sigma ^{(\ell )}_{r_\ell }\ge \tau \sigma _1^{(\ell )}>\sigma ^{(\ell )}_{r_\ell + 1}, \end{aligned}$$

where the low rank factors are

17 $$\begin{aligned} {\mathsf W}^{(\ell )} = \left[ \begin{array}{ccc} {\boldsymbol{u}}^{(\ell )}_1&\cdots&{\boldsymbol{u}}^{(\ell )}_{r_\ell }\end{array}\right] , \quad {\mathsf H}^{(\ell )} = \left[ \begin{array}{c} \sigma _1^{\ell )}\big ({\boldsymbol{v}}^{(\ell )}_1\big )^{\mathsf T}\\ \vdots \\ \sigma _{r_\ell }^{(\ell )}\big ({\boldsymbol{v}}^{(\ell )}_{r_\ell }\big )^{\mathsf T}\end{array}\right] . \end{aligned}$$

The low-rank approximation is effectively a subdictionary compression. We refer to the columns of ${\mathsf W}^{(\ell )}$ as the feature vectors, and to ${\mathsf W}^{(\ell )}$ as the code book, of the *ℓ*th subdictionary. We write

18 $$\begin{aligned} {\mathsf D}^{(\ell )} = {\mathsf W}^{(\ell )} {\mathsf H}^{(\ell )} + {\mathsf E}^{(\ell )}, \end{aligned}$$

where the matrix ${\mathsf E}^{(\ell )}$ accounts for the compression error. We point out that while the low rank approximation as outlined above was based on the SVD, the considerations about dictionary compression hold regardless of how the low rank approximation is obtained. Repeating the procedure for all subdictionaries, we have

19 $$\begin{aligned} {\mathsf D}= & \left[ \begin{array}{cccc} {\mathsf D}^{(1)}&{\mathsf D}^{(2)}&\cdots&{\mathsf D}^{(L)} \end{array}\right] \end{aligned}$$

20 $$\begin{aligned}= & \left[ \begin{array}{cccc} {\mathsf W}^{(1)} {\mathsf H}^{(1)}&{\mathsf W}^{(2)}{\mathsf H}^{(2)}&\cdots&{\mathsf W}^{(L)} {\mathsf H}^{(L)} \end{array} \right] + \left[ \begin{array}{cccc} {\mathsf E}^{(1)}&{\mathsf E}^{(2)}&\cdots&{\mathsf E}^{(L)} \end{array}\right] \end{aligned}$$

21 $$\begin{aligned}= & {\mathsf W}{\mathsf H}+ {\mathsf E}, \end{aligned}$$

where

22 $$\begin{aligned} {\mathsf W}= \begin{bmatrix} \begin{array}{ccc} {\mathsf W}^{(1)}&\dots&{\mathsf W}^{(L)} \end{array} \end{bmatrix}, \quad {\mathsf H}= \begin{bmatrix} {\mathsf H}^{(1)} & 0 & \dots & 0 \\ 0 & {\mathsf H}^{(2)} & 0 & \vdots \\ \vdots & 0 & \ddots & 0 \\ 0 & \dots & 0 & {\mathsf H}^{(L)} \end{bmatrix}. \end{aligned}$$

The matrix ${\mathsf W}$ is a compression of the dictionary ${\mathsf D}$. The goal of Phase I of the algorithm is to approximate a given input ${\boldsymbol{y}}$ in terms of the atoms of ${\mathsf W}$, which are the feature vectors of all the subdictionaries.

### Phase I: Identification of Relevant Subdictionaries

To formulate the compressed dictionary matching problem (11) as an inverse problem in the Bayesian framework, we need to define a likelihood model, a prior model, and discuss how the resulting posterior distribution is used for finding a feasible estimate.

#### Likelihood Model

For the likelihood model, we write a stochastic model in terms of random variables,

23 $$\begin{aligned} {\boldsymbol{Y}}= {\mathsf W}{\boldsymbol{Z}}+ {\boldsymbol{E}}+ {\boldsymbol{S}}, \end{aligned}$$

where ${\boldsymbol{Y}}$ is a random variable representing the input, ${\boldsymbol{Z}}$ is a random variable of the dictionary coefficients, ${\boldsymbol{E}}$ represents the *dictionary compression error (DCE)*, and ${\boldsymbol{S}}$ accounts for the noise in the data. This model is the basis of the likelihood model of the problem.

To analyze the DCE, consider an arbitrary dictionary atom ${\boldsymbol{d}}_k$, the *k*th column of ${\mathsf D}$, and find its best approximation ${\mathsf W}{\boldsymbol{z}}_k$ in terms of the compressed dictionary,

24 $$\begin{aligned} {\boldsymbol{z}}_k = {\mathop {\mathrm{argmin}}\limits _{{\boldsymbol{z}}}}\Vert {\boldsymbol{d}}_k - {\mathsf W}{\boldsymbol{z}}\Vert . \end{aligned}$$

We decompose the atoms as

25 $$\begin{aligned} {\boldsymbol{d}}_k= & {\mathsf W}{\boldsymbol{z}}_k + ({\boldsymbol{d}}_k - {\mathsf W}{\boldsymbol{z}}_k )\end{aligned}$$

26 $$\begin{aligned}= & {\mathsf W}{\boldsymbol{z}}_k + {\boldsymbol{e}}_k, \quad 1 \le k \le N, \end{aligned}$$

where the vector ${\boldsymbol{e}}_k$,

27 $$\begin{aligned} {\boldsymbol{e}}_k = {\boldsymbol{d}}_k - {\mathsf W}{\boldsymbol{z}}_k, \end{aligned}$$

represents only the dictionary compression error. To encode the compression error in terms of a probability density, we assume that the vectors ${\boldsymbol{e}}_k$ are independent realizations of the DCE random variable ${\boldsymbol{E}}$, and use a Gaussian approximation for its probability distribution,

28 $$\begin{aligned} {\boldsymbol{E}}\sim \mathcal {N}({\boldsymbol{\mu }}_\textrm{DCE}, {\mathsf C}_\textrm{DCE}). \end{aligned}$$

where the mean and covariance are estimated from the sample,

29 $$\begin{aligned} {\boldsymbol{\mu }}_\textrm{DCE}= & \frac{1}{N} \sum {\boldsymbol{e}}_k, \end{aligned}$$

30 $$\begin{aligned} {\mathsf C}_\textrm{DCE}= & \frac{1}{N-1} \sum ({\boldsymbol{e}}_k - {\boldsymbol{\mu }}_\textrm{DCE}) ({\boldsymbol{e}}_k - {\boldsymbol{\mu }}_\textrm{DCE})^{\mathsf T}. \end{aligned}$$

Assuming that the measurement error ${\boldsymbol{S}}$ is independent of the compression error, and modeling it as scaled white noise,

31 $$\begin{aligned} {\boldsymbol{S}}\sim {\mathcal {N}}(0, \delta ^2 {\mathsf I}_m), \end{aligned}$$

where *δ>0* represents the noise level, the likelihood model for ${\boldsymbol{Y}}$ based on (23), conditional on ${\boldsymbol{Z}}={\boldsymbol{z}}$ is of the form

32 $$\begin{aligned} \pi _{{\boldsymbol{Y}}\mid {\boldsymbol{Z}}}({\boldsymbol{y}}\mid {\boldsymbol{z}}) \propto \textrm{exp}\left( -\frac{1}{2} \Vert {\mathsf C}^{-1/2}({\mathsf W}{\boldsymbol{z}}+ {\boldsymbol{\mu }}_{DCE} - {\boldsymbol{y}})\Vert ^2 \right) , \end{aligned}$$

where

33 $$\begin{aligned} {\mathsf C}= {\mathsf C}_\textrm{DCE} + \delta ^2 {\mathsf I}_m. \end{aligned}$$

For later reference, we write the model (23) in terms of the compressed subdictionaries as

34 $$\begin{aligned} {\boldsymbol{Y}}= \sum _{\ell =1}^L{\mathsf W}^{(\ell )}{\boldsymbol{Z}}^{(\ell )} +{\boldsymbol{E}}+{\boldsymbol{S}}, \end{aligned}$$

where ${\boldsymbol{Z}}^{(\ell )}\in {\mathbb {R}}^{r_\ell }$ is a random variable containing the coefficients associated to the *ℓ*th code book.

#### Prior Model

To promote an approximate representation of the input ${\boldsymbol{y}}$ in terms of few compressed dictionaries, we introduce a prior that favors solutions in which only few of the coefficient vectors ${\boldsymbol{Z}}^{(\ell )}$ in (34) are non-zero. To this end, we start by introducing a zero-mean conditionally Gaussian prior model,

35 $$\begin{aligned} {\boldsymbol{Z}}^{(1)},\ldots {\boldsymbol{Z}}^{(L)} \text{ mutually } \mathrm{ independent, } {\boldsymbol{Z}}^{(\ell )} \sim {\mathcal {N}}(0,\theta _\ell {\mathsf G}_\ell )\text{, } \end{aligned}$$

where the matrices ${\mathsf G}_\ell \in {\mathbb {R}}^{r_\ell \times r_\ell }$ will be defined below, and $\theta _\ell>0$ are scaling parameters. Assuming that ${\mathsf G}_\ell$ and $\theta _\ell$ are given, the prior probability density assumes the form

36 $$\begin{aligned}&\pi _{\boldsymbol{Z}}(z) \propto \frac{1}{|\textrm{det}(\theta _1{\mathsf G}_1)|^{1/2} \cdots |\textrm{det}(\theta _1{\mathsf G}_1)|^{1/2}} \times\nonumber \\& \textrm{exp} \left( -\frac{1}{2} \sum _{\ell =1}^L \frac{({\boldsymbol{z}}^{(\ell )})^{\mathsf T}{\mathsf G}_\ell ^{-1}{\boldsymbol{z}}^{(\ell )}}{\theta _\ell } \right) \end{aligned}$$

37 $$\begin{aligned}&\propto \textrm{exp}\left( -\frac{1}{2} \sum _{\ell =1}^L \frac{({\boldsymbol{z}}^{(\ell )})^{\mathsf T}{\mathsf G}_\ell ^{-1}{\boldsymbol{z}}^{(\ell )}}{\theta _\ell } \right. \nonumber \\&\left. - \frac{1}{2} \sum _{\ell =1}^L r_\ell \log \theta _\ell \right) , \end{aligned}$$

where the dependency of the determinants on the parameters $\theta _\ell$ is included in the exponential, while the determinants of the matrices ${\mathsf G}_\ell$ are ignored.

In the current problem, the goal is to find a group-sparse solution ${\boldsymbol{z}}$ such that the size of most of the vectors ${\boldsymbol{z}}^{(\ell )}$ is insignificant. In the prior (37), the parameter $\theta _\ell$ plays the role of the variance of ${\boldsymbol{z}}^{(\ell )}$, however we do not know a priori which of the parameters $\theta _\ell$ should take on a negligible value, therefore in line with the Bayesian paradigm, we model the $\theta _\ell$ as random variables. As explained in the Appendix, an effective way to let the values of these parameters be guided by the data, is to assume that the corresponding random variables $\Theta _\ell$ are mutually independent and distributed according to Generalized Gamma probability density of the form

38 $$\begin{aligned} \Theta _\ell \sim \theta ^{r\beta _\ell -1}\textrm{exp}\left( - \left( \frac{\theta _\ell }{\vartheta _j}\right) ^r\right) \nonumber \\ =\textrm{exp} \left( - \left( \frac{\theta _\ell }{\vartheta _\ell }\right) ^r +(r\beta _\ell - 1)\log \theta _\ell \right) , \end{aligned}$$

where $\beta _\ell>0$ and *r≠ 0* are shape parameters, and $\vartheta _\ell>0$ are scaling parameters. We therefore interpret the prior model (37) as a conditional distribution of ${\boldsymbol{Z}}$ given ${\boldsymbol{\Theta }}= {\boldsymbol{\theta }}= (\theta _1,\ldots ,\theta _L)$, and extend the prior model by writing

39 $$\begin{aligned}&\pi _{{\boldsymbol{Z}},{\boldsymbol{\Theta }}}({\boldsymbol{z}},{\boldsymbol{\theta }}) = \pi _{{\boldsymbol{Z}}\mid {\boldsymbol{\Theta }}}({\boldsymbol{z}}\mid {\boldsymbol{\theta }})\pi _\Theta ({\boldsymbol{\theta }})\end{aligned}$$

40 $$\begin{aligned}&\propto \textrm{exp}\left( -\frac{1}{2} \sum _{\ell =1}^L \frac{({\boldsymbol{z}}^{(\ell )})^{\mathsf T}{\mathsf G}_\ell ^{-1}{\boldsymbol{z}}^{(\ell )}}{\theta _\ell } - \sum _{\ell =1}^L\left( \frac{\theta _\ell }{\vartheta _\ell }\right) ^r \right. \nonumber \\&\left. -\sum _{\ell =1}^L\left( \frac{r_\ell }{2} +1 -r\beta _\ell \right) \log \theta _\ell \right) . \end{aligned}$$

To complete the description of the prior model, we need to define the matrices ${\mathsf G}_\ell \in {\mathbb {R}}^{r_\ell \times r_\ell }$. Consider the matrix ${\mathsf H}^{(\ell )} \in {\mathbb {R}}^{r_\ell \times n_\ell }$ in the low-rank approximation (18) of ${\mathsf D}^{(\ell )}$, where $n_\ell$ is the number of atoms in the *ℓ*th subdictionary. In view of the likelihood model (34), the columns of ${\mathsf H}^{(\ell )}$ can be considered to represent typical coefficient vectors for matching data from the *ℓ*th subdictionary in terms of the code book ${\mathsf W}^{(\ell )}$. However, since we do not know a priori if the *ℓ*th subdictionary is relevant for the input vector ${\boldsymbol{z}}$, and in fact we expect most subdictionaries to not be relevant, we extract from ${\mathsf H}^{(\ell )}$ only information about the direction of its columns. To that end, consider the the lean SVD of the matrix ${\mathsf H}^{(\ell )}$,

41 $$\begin{aligned} {\mathsf H}^{(\ell )}= {\mathsf Q}^{(\ell )} {\mathsf \Lambda }^{(\ell )} \big [{\mathsf P}^{(k)}\big ]^{\mathsf T}, \quad {\mathsf \Lambda }^{(\ell )} = \textrm{diag}\big ([\lambda ^{(\ell )}_1,\ldots ,\lambda ^{(\ell )}_{p_\ell }]\big ), \end{aligned}$$

where $p_\ell \le r_\ell$ is the number of non-zero singular values, and denote the left singular vectors corresponding to the non-zero singular values as ${\boldsymbol{q}}_j$,

42 $$\begin{aligned} {\mathsf Q}^{(\ell )} = \left[ \begin{array}{ccc} {\boldsymbol{q}}_1&\cdots&{\boldsymbol{q}}_{p_\ell }\end{array}\right] . \end{aligned}$$

**Fig. 2:**
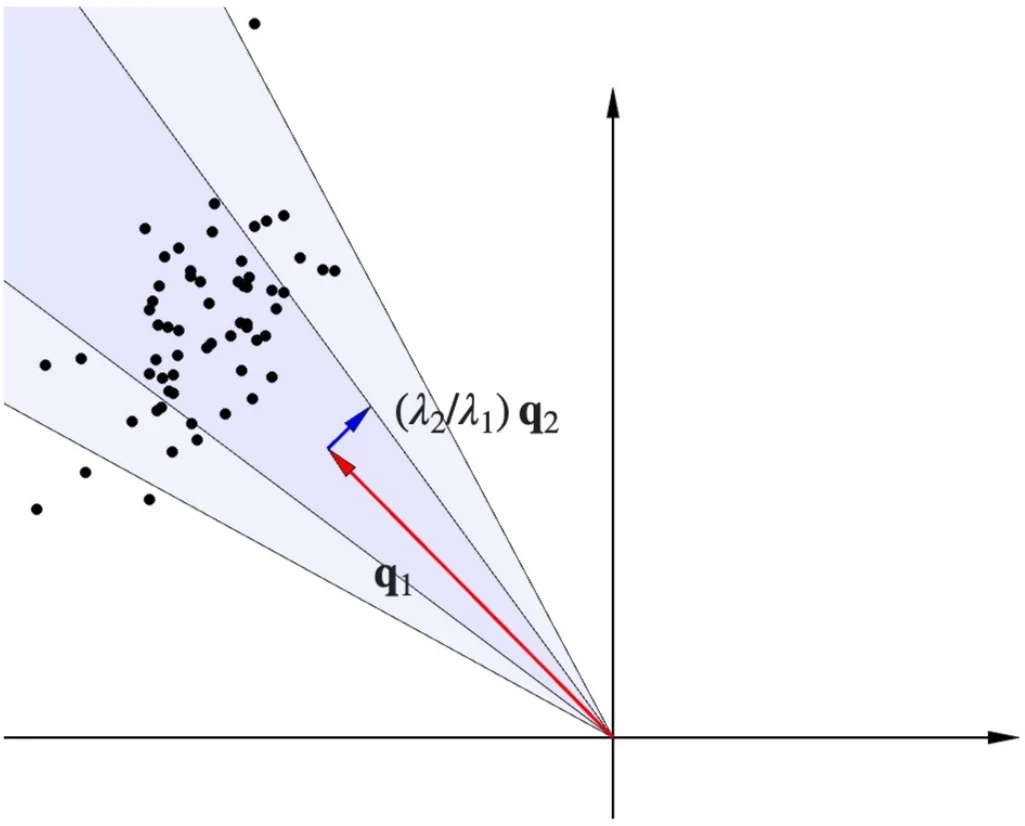
Schematic two-dimensional figure explaining the prior design. The position vectors of the points indicated in the figure correspond to the column vectors of the matrix ${\mathsf H}^{(\ell )}$. The vectors ${\boldsymbol{q}}_1$ and ${\boldsymbol{q}}_2$ are the two normalized left singular vectors of the matrix ${\mathsf H}^{(\ell )}$ and $\lambda _1$ and $\lambda _2$ the corresponding singular values. The shaded regions indicate the cones of one and two standard deviations (68.3% and 95.5% probabilities, respectively) of the direction-dependent prior distribution defined by the matrix ${\mathsf G}_\ell$, formula (43) with *\varepsilon = 0*. The ${\mathsf G}_\ell$ weighted squared norm $\Vert {\boldsymbol{z}}\Vert _{{\mathsf G}_\ell }^2 = {\boldsymbol{z}}^{\mathsf T}{\mathsf G}_\ell ^{-1}{\boldsymbol{z}}$ appearing in the definition (36) penalizes vectors for deviating from the preferred directions defined by the columns of ${\mathsf H}^{(\ell )}.$

Interpreting the columns of the matrix ${\mathsf H}^{(\ell )}$ as a cloud of $n_\ell$ points in the space ${\mathbb {R}}^{r_\ell }$, the first singular vector ${\boldsymbol{q}}_1$ points in the direction in ${\mathbb {R}}^{r_\ell }$ of the center of mass of the point cloud, while the subsequent singular vectors ${\boldsymbol{q}}_j, \; j>1$ point in the orthogonal directions where the point cloud has a spread proportional to the singular values $\big (\lambda _j^{(\ell )}\big )^2$, see Fig. 2. Inspired by this geometric observation, we define the prior covariance matrix ${\mathsf G}_\ell$ as

43 $$\begin{aligned} {\mathsf G}_\ell= & \left( \frac{1}{\lambda _1^{(\ell )}}\right) ^2 {\mathsf Q}^{(\ell )} {\mathsf \Lambda }^{(\ell )} \big [{\mathsf \Lambda }^{(\ell )}\big ]^{\mathsf T}\big [{\mathsf Q}^{(\ell )}\big ]^{\mathsf T}+ \epsilon \, {\mathsf I}_{r_\ell } \end{aligned}$$

44 $$\begin{aligned}= & {\boldsymbol{q}}_1 {\boldsymbol{q}}_1^{\mathsf T}+ \sum _{j=2}^{p_\ell } \kappa _j{\boldsymbol{q}}_j {\boldsymbol{q}}_j^{\mathsf T}+ \varepsilon \, {\mathsf I}_{r_\ell }, \quad \kappa _j = \left( \frac{\lambda _j^{(\ell )}}{\lambda _1^{(\ell )}}\right) ^2, \end{aligned}$$

where *\varepsilon>0* is a small parameter and $\varepsilon \, {\mathsf I}_{r_\ell }$ is a regularization term added to guarantee positive definiteness. Denoting by $\widehat{\boldsymbol{z}}^{(\ell )}$ the unit vector collinear with ${\boldsymbol{z}}^{(\ell )}$, we may write

45 $$\begin{aligned}&\frac{({\boldsymbol{z}}^{(\ell )})^{\mathsf T}{\mathsf G}_\ell ^{-1}{\boldsymbol{z}}^{(\ell )}}{\theta _\ell } = \frac{\Vert {\boldsymbol{z}}^{(\ell )}\Vert ^2}{\theta _\ell } \left( \frac{1}{1+\varepsilon } ({\boldsymbol{q}}_1^{\mathsf T}\widehat{\boldsymbol{z}}^{(\ell ))})^2 \right. \nonumber \\&\left. + \sum _{j=2}^{p_\ell }\frac{1}{\kappa _j+\varepsilon } ({\boldsymbol{q}}_j^{\mathsf T}\widehat{\boldsymbol{z}}^{(\ell ))})^2 +\frac{1}{\varepsilon }\Vert {\mathsf P}^\perp \widehat{\boldsymbol{z}}^{(\ell )}\Vert ^2 \right) , \end{aligned}$$

where ${\mathsf P}^\perp$ is the orthogonal projection onto the subspace perpendicular to the range of ${\mathsf H}^{(\ell )}$. In this prior model, the amplitude $\Vert {\boldsymbol{z}}^{(\ell )}\Vert$ of the vector is scaled by the reciprocal of the parameter $\theta _\ell$, while the second factor penalizes deviation of the direction away from the preferred directions of the columns of ${\mathsf H}^{(\ell )}$.

#### The Posterior and the MAP Estimate

Combining likelihood and prior according to Bayes’ formula yields a posterior model for the pair $({\boldsymbol{Z}},{\boldsymbol{\Theta }})$ of the form

46 $$\begin{aligned}&\pi _{{\boldsymbol{Z}},{\boldsymbol{\Theta }}\mid {\boldsymbol{Y}}}({\boldsymbol{z}},{\boldsymbol{\theta }}\mid {\boldsymbol{y}}) \propto \pi _{{\boldsymbol{Y}}\mid {\boldsymbol{Z}}}({\boldsymbol{y}}\mid {\boldsymbol{z}})\pi _{{\boldsymbol{Z}},{\boldsymbol{\Theta }}}({\boldsymbol{z}},{\boldsymbol{\theta }}) \end{aligned}$$

47 $$\begin{aligned}&\propto \textrm{exp}\left( -\frac{1}{2} \Vert {\mathsf C}^{-1/2}({\mathsf W}{\boldsymbol{z}}\right. \nonumber \\&\left. + {\boldsymbol{\mu }}_{DCE} - {\boldsymbol{y}})\Vert ^2 -\frac{1}{2}\sum _{\ell =1}^L\frac{({\boldsymbol{z}}^{(\ell )})^{\mathsf T}{\mathsf G}_\ell ^{-1}{\boldsymbol{z}}^{(\ell )}}{\theta _\ell } - \Phi ({\boldsymbol{\theta }}) \right) , \end{aligned}$$

where

48 $$\begin{aligned} \Phi ({\boldsymbol{\theta }}) = \sum _{\ell =1}^L\left( \frac{\theta _\ell }{\vartheta _\ell }\right) ^r + \sum _{\ell =1}^L\left( \frac{r_\ell }{2} +1 -r\beta _\ell \right) \log \theta _\ell . \end{aligned}$$

The structure of the posterior density is well suited for the IAS algorithm, an efficient alternating scheme for computing a maximum a posteriori (MAP) estimate, extensively discussed in the literature (Calvetti et al. 2023), and briefly reviewed in the Appendix. The IAS algorithm can be summarized as follows:

1. Initialize ${\boldsymbol{\theta }}^0 = {\boldsymbol{\vartheta }}$, setting the counter *ν =0*.
2. Repeat until a convergence criterion is met:
  1. Given the current value ${\boldsymbol{\theta }}^\nu$ update ${\boldsymbol{z}}= {\boldsymbol{z}}^{\nu +1}$ by minimizing
    49 $$\begin{aligned}&E_{\boldsymbol{\theta }}({\boldsymbol{z}}) = \frac{1}{2} \Vert {\mathsf C}^{-1/2}({\mathsf W}{\boldsymbol{z}}+ {\boldsymbol{\mu }}_{DCE} - {\boldsymbol{y}})\Vert ^2 \nonumber \\&+\frac{1}{2} \sum _{\ell =1}^L\frac{({\boldsymbol{z}}^{(\ell )})^{\mathsf T}{\mathsf G}_\ell ^{-1}{\boldsymbol{z}}^{(\ell )}}{\theta _\ell }, \quad {\boldsymbol{\theta }}= {\boldsymbol{\theta }}^\nu . \end{aligned}$$
  2. Given the current value ${\boldsymbol{z}}={\boldsymbol{z}}^{\nu +1}$ update ${\boldsymbol{\theta }}= {\boldsymbol{\theta }}^{\nu +1}$ by minimizing
    50 $$\begin{aligned} E_{\boldsymbol{z}}({\boldsymbol{\theta }}) =\frac{1}{2} \sum _{\ell =1}^L \frac{({\boldsymbol{z}}^{(\ell )})^{\mathsf T}{\mathsf G}_\ell ^{-1}{\boldsymbol{z}}^{(\ell )}}{\theta _\ell } + \Phi ({\boldsymbol{\theta }}), \quad {\boldsymbol{z}}= {\boldsymbol{z}}^{\nu +1}. \end{aligned}$$
  3. Advance the counter $\nu \rightarrow \nu +1$ and check the convergence condition.

The efficiency of the IAS algorithm, compared, e.g., with $\ell ^1$-based penalty functionals for promoting sparsity, arises from the fact that the updating of ${\boldsymbol{z}}$ only requires the solution of a quadratic least squares problem, while the updating of ${\boldsymbol{\theta }}$ can be performed component-wise, and the one-dimensional minimization problems for each component are simple to implement, in some cases admitting an explicit closed-form formula. We refer to Calvetti and Somersalo (2023) for a general discussion of the IAS algorithm for different choices of hyperpriors from the generalized Gamma distribution, the parameters $\beta _k$ and $\vartheta _k$, and to Calvetti and Somersalo (2025, 2025) for recent computational developments. To overcome the non-convexity of the optimization problem when *r<1*, a hybrid version of the algorithm was proposed. The hybrid scheme starts the iteration with the gamma hyperprior (*r=1*), characterized by guaranteed convexity of the objective function and convergence of the IAS algorithm to the unique minimizer, and at some point switches to a hyperprior with *r<1* leading to fast local convergence with stronger sparsity promotion, see, e.g., Calvetti et al. (2020a). As pointed out in the cited articles, the choices of the values of the other parameters $\beta _j$ and $\vartheta _j$ are related to the sparsity and sensitivity, and detailed out when the computed examples are described.

### Dictionary Deflation

In the numerical tests, we will consider the performance of the Phase I only as a classifier: using the winner-takes-all criterion, we classify the activity as coming from the brain region with the maximum $\theta _\ell$. Using the IAS algorithm to compute the Maximum A Posteriori (MAP) estimate for the pair $(\textbf{z},{\boldsymbol{\theta }})$,

51 $$\begin{aligned} (\textbf{z}^*,{\boldsymbol{\theta }}^*) = \underset{(\textbf{z},{\boldsymbol{\theta }})}{\textrm{argmax}}\, \pi _{\textbf{Z},{\boldsymbol{\Theta }}\mid {\textbf{Y}}}(\textbf{z},{\boldsymbol{\theta }}\mid {\textbf{y}}), \end{aligned}$$

the brain regions where the activity behind the observation is supported can be identified by monitoring the parameter ${\boldsymbol{\theta }}$: The active regions are those associated with the most significant values, i.e., the regions for which

52 $$\begin{aligned} \theta _j> p\,\max _{1\le \ell \le L}(\theta _\ell ), \end{aligned}$$

where *p<1* is a threshold parameter, e.g., *p =0.005*, are the most likely support of the activation underneath the MEG signal.

While the phase I of the dictionary learning algorithm described above leads reliably to a compressible solution, with most of the values of the parameter $\theta _\ell$ negligible, using that information to find a single brain region according to the winner-takes-all criterion described above may fail, because occasionally, the value of $\theta _\ell$ for the activated brain region is relatively large, but not necessarily the largest. As shown in Bocchinfuso et al. (2024), the success of classification algorithm described above can be improved significantly by using the results of Phase I for detecting the relevant subdictionaries and performing a sparse dictionary coding step with the deflated dictionary. More precisely, once the MAP estimate (51) has been computed, the subset of indices

53 $$\begin{aligned} I_\textrm{defl}(p) = \{\ell \mid \theta _\ell> p\, \max (\theta _{i})\}, \;0<p<1, \end{aligned}$$

where *p* is a threshold parameter, identifies the few brain regions that the compression algorithm deemed relevant for explaining the data, defining the reduced dictionary

54 $$\begin{aligned} {\mathsf D}^\textrm{defl} = \left[ \begin{array}{ccc} {\mathsf D}^{(\ell _1)}&\cdots&{\mathsf D}^{(\ell _q)}\end{array}\right] , \quad I_\textrm{defl}(p) =\{\ell _1,\ldots ,\ell _q\}. \end{aligned}$$

### Phase II: Best Explaining Dipole Identification

The second phase in the classification algorithm looks for an optimal representation of the data vector in terms of a few atoms of the reduced dictionary,

55 $$\begin{aligned} {{\boldsymbol{y}}} = {\mathsf D}^\textrm{defl} {{\boldsymbol{x}}} + {{\boldsymbol{e}}}', \quad {{\boldsymbol{x}}} \text{ sparse, } \end{aligned}$$

where the vector ${{\boldsymbol{e}}}'$ represents the observation uncertainty in the data, by minimizing the discrepancy while promoting sparsity for the vector ${{\boldsymbol{x}}}$, using the sparsity promoting IAS algorithm described in the Appendix.

Having computed the coefficient vector ${\boldsymbol{x}}$, a winner-takes-all classification strategy is employed to associate the data ${{\boldsymbol{y}}}$ with the subdictionary in $I_\textrm{defl}(p)$ that contributes most to its explanation. The criterion for the best match is done similarly as in the first phase, by identifying the component with maximal variance in a Bayesian hierarchical model.

An advantage of the two-phase algorithm is that the first phase is more robust and less sensitive to noise than a sparsity-based dictionary matching with the full dictionary, as the feature vectors summarizing the subdirectories typically are not a good match for noise. The lack of fine details in the compressed dictionary is compensated for in the second phase, where the dictionary matching problem is solved again with a reduced dictionary obtained by removing those atoms deemed irrelevant in the first phase. The computational efficiency of this algorithm stems from the fact that both phases work with significantly reduced dictionaries.

## Performance Assessment Methods

Typically the performance of dictionary classifiers are tested by on a data sets consisting of inputs with known class label, ${\mathscr {T}}_\textrm{test} = \big \{({\boldsymbol{y}}^{(k)},c_k)\big \}_{k=1}^K$. In the brain activity identification from MEG data, the testing will be done on simulated data, because of the general unavailability of ground truth with experimental data. Here, we limit the discussion to simulated activity in a single brain region, details being discussed with the computed examples.

### Confusion Matrices and Impurity Indices

Denote by ${\mathsf C}$ the *L× L* confusion matrix, where the value of ${\mathsf C}_{ij}$ is the number of times when class *j* has been assigned to data arising from an activation in class *i*. A row-wise interpretation of the confusion matrix, with the rows suitably normalized, gives the frequency at which each active brain region is assigned to data with a given label. Particularly interesting from the point of view of the inverse problem of region identification is the column-wise interpretation. The entries of the *j*th column of ${\mathsf C}$ scaled by its 1-norm,

56 $$\begin{aligned} {\mathsf P}_{ij} = \frac{{\mathsf C}_{ij}}{\sum _{i'=1}^L {\mathsf C}_{i'j},} \quad \text{ j } \text{ given, } \end{aligned}$$

indicate the frequencies of the activation having occurred in regions *i*, given that the algorithm has identified the *j*th region as the winner. Therefore we can interpret the entries of the column-normalized confusion matrix as conditional probabilities,

57 $$\begin{aligned} {\mathsf P}_{ij} = P(\text{ region } \text{ i } \text{ is } \text{ active }\mid \text{ region } \text{ j } \text{ was } \text{ identified}), \quad 1\le j\le N. \end{aligned}$$

Similarly, the entries of the row-normalized confusion matrix,

58 $$\begin{aligned} {\mathsf Q}_{ij} = \frac{{\mathsf C}_{ij}}{\sum _{j'=1}^L {\mathsf C}_{i j'},}\quad \text{ i } \text{ given, } \end{aligned}$$

admit the conditional probability interpretation,

59 $$\begin{aligned} {\mathsf Q}_{ij} = P(\text{ region } \text{ j } \text{ is } \text{ identified }\mid \text{ region } \text{ i } \text{ is } \text{ active}), \quad 1\le j\le N. \end{aligned}$$

The matrix ${\mathsf P}$ can be thought of as a generalization of *recall* defined for binary classifiers, and the matrix ${\mathsf Q}$ as a generalization of the *precision*.

To assess the performance of each phase separately, we denote by ${\mathsf C}^1$ the confusion matrix corresponding to the compressed dictionary step with the winner-takes-all classification, referred to as Phase I, and by ${\mathsf C}^2$ the confusion matrix after the completion of the deflated dictionary step, referred to as Phase II, and we use the notations ${\mathsf P}^1$ and ${\mathsf P}^2$ for the respective column-normalized versions.

Borrowing from the data science methodology, the performance of the algorithm associated to a specific brain region can be quantified by different impurity indices. Given the matrix ${\mathsf P}$, the misclassification rate $MCR_j$ gives the probability that the proposed classification to the *j*th class is incorrect,

60 $$\begin{aligned} MCR_j = \sum _{i\ne j}^L P_{ij} = 1 - P_{jj}. \end{aligned}$$

While the *MCR* is a good measure for the success rate, it is insensitive to the spread of the erroneous classifications. More informative measures of the spread are the Gini index $G_j$ and the cross entropy $S_j$,

61 $$\begin{aligned} G_j =\sum _{i=1}^LP_{ij}(1-P_{ij}),\quad S_j =-\sum _{i=1}^L P_{ij}\log P_{ij}. \end{aligned}$$

We will use these impurity measures to assess the performance of the algorithm, as well as to assess to what extent the second phase improves/deteriorates the classification results.

### Identification Trees

The column-normalized confusion matrix ${\mathsf P}$ suggests an alternative interpretation of the probability of the activity attributed to a given ROI as a directed network. More specifically, each column of ${\mathsf P}$ can be associated with a weighted directed tree graph with a self-loop at the root, which is the node associated with the ROI indexing the column. The remaining nodes are the other ROIs in the atlas and the entries of the column are the weights of the edges from the root node. The complexity of the trees depends on how well the root ROI is identifiable: for most ROIs the tree has only a few leaves , and only very few ROIs are associated with a tree with many leaves.

Recalling that anatomical considerations have been the guiding principle behind the Destrieux atlas, and in view of the tight packing of gyri and sulci, it is not unreasonable that some of ROIs are very difficult to identify unambiguouslyfrom MEG data. The location of a ROI relative to the placement of the sensors measuring the magnetic field, its volume and its positions in relation to its neighbors are factors playing an important role in how well it can be recovered. We demonstrate this idea with computed examples in Sect. 6.

## Methods: Simulation and Assessment of Performance

In this section we test how well the brain regions defined by the Destrieux atlas can be identified from MEG data with the proposed Bayesian dictionary learning methodology by means of the following numerical protocol. In addition to assessing the success of the proposed algorithms as a classifier, the results of the simulations open a discussion on whether brain atlases based on anatomical brain features should be replaced by brain atlases focusing on the cerebral electromagnetic activity for studies based on MEG and EEG data, e.g., by identifying brain regions that are unambiguously identifiable by the available data.

**Forward model:** We download the head model and the corresponding lead field matrices ${\mathsf L}_k \in {\mathbb {R}}^{m\times 3}$, $1\le k\le N$ from the Open MEG Archive (OMEGA) website https://www.mcgill.ca/bic/neuroinformatics/omega, see Niso et al. (2016). The discretized head model consist of *N = 15  002* dipoles, and we fix the dipole orientations parallel to the normal vector of the local cortical surface, estimated from the anatomical MRI data as indicated in the cited article.The lead field matrix corresponds to *m=306* measurements performed by 102 sensors, each comprising a magnetometer and two gradiometers, recording the magnetic flux density as well as two tangential gradient components. The source space is divided into *L = 148* parcels according to the Destrieux brain atlas (Destrieux et al. 2010) using the automatic parcellation provided by Brainstorm, see Tadel et al. (2011), constituting the basis for the partition into subdictionaries.

**Source simulation:** For each brain region, we generate 100 activity patterns by first selecting a random source space point within the region, and then identifying five nearest nodes within the same region. In each of these positions, we place a random dipole parallel to the vectors normal to the cortex at that point, and draw the amplitude from the uniform distribution over the interval [0, 1]. The noiseless magnetometer/gradiometer data corresponding to the patch activity is computed, and scaled Gaussian white noise (i.e., independent in each channel) with standard deviation *σ = 0.005*) is added.

**Assessment pipeline:** Starting with the simulated noisy MEG data, we run the two-phase classifier, originally proposed in Bocchinfuso et al. (2024).

- **Phase I:** The first phase is based on the hybrid group sparsity IAS algorithm, in which the MAP minimization for the compressed dictionary is first estimated using the gamma hyperprior (*r=1*) that is guaranteed to converge to a unique global minimum, then switching to generalized gamma hyperprior model with the model parameter *r=1/2* in (38) to more strongly promote sparsity in the solution and to speed up the convergence (see Appendix). The process is repeated 100 times, one for each simulation, for every brain region, resulting in a total of 14 800 classification tests. We tally the winner-takes-all classification results in the confusion matrices ${\mathsf C}^1$.
- **Dictionary compression:** At the end of Phase I for each simulation, we discard all dipoles of the full dictionary in regions not satisfying (52) value *p = 0.005*.
- **Phase II:** The second phase solves the deflated dictionary matching problem using the IAS algorithm with the gamma hyperprior model, *r=1*. The algorithm converges fast to the unique minimum identifying the few best explaining dipoles. As in Phase I, we repeat this process for each simulation and tally the winner-takes-all classification results of Phase II, storing the results in the confusion matrix ${\mathsf C}^2$.

**Model scaling:** The dictionary learning interpretation differs slightly from the traditional MEG inverse problem setting in the way the model is scaled. Starting with the forward model (2) with additive noise,

62 $$\begin{aligned} {\boldsymbol{b}}= \sum _{j=1}^N {\mathsf L}_k {\boldsymbol{q}}_k + {\boldsymbol{\epsilon }}, \end{aligned}$$

we write the scaled model as

$$\begin{aligned} & {\boldsymbol{y}}= \frac{{\boldsymbol{b}}}{\Vert {\boldsymbol{b}}\Vert } = \sum _{j=1}^N \underbrace{\left\{ \frac{\pm \Vert {\boldsymbol{q}}_k\Vert \Vert {\mathsf L}_k\widehat{\boldsymbol{q}}_k\Vert }{\Vert {\boldsymbol{b}}\Vert }\right\} }_{x_k} \underbrace{\left\{ \frac{{\mathsf L}_k\widehat{\boldsymbol{q}}_k}{\Vert {\mathsf L}_k\widehat{\boldsymbol{q}}_k\Vert }\right\} }_{{\boldsymbol{d}}_k} + \frac{{\boldsymbol{\epsilon }}}{\Vert {\boldsymbol{b}}\Vert } \\ & = \sum _{j=1}^N{\boldsymbol{d}}_k x_k + {\boldsymbol{\eta }} ={\mathsf D}{{\boldsymbol{x}}} + {\boldsymbol{\eta }}. \end{aligned}$$

Assuming that the signal-to-noise-ratio (SNR) of the data is given, we may assume that the variance of ${\boldsymbol{\eta }}$ is given for all simulations. This justifies the choice of the noise model in the simulations, corresponding to SNR of the order of $4.6\,\textrm{dB}$. The fact that the columns of the matrix ${\mathsf D}$ are scaled to unity implies that *y* is equally sensitive to each $x_k$, hence the algorithm does not favor superficial sources over deep sources. While the scaling by the norm of ${\boldsymbol{b}}$ and the column norms of ${\mathsf D}$ have an effect on the values $x_k$, in this approach we are not interested in the source amplitude, but only in the support of the source. As pointed out in the Appendix, the column scaling of the matrix is in line with the Bayesian hypermodel, removing the need to compensate for the variable sensitivity of the model to different components of the unknown.

## Results

**Fig. 3:**
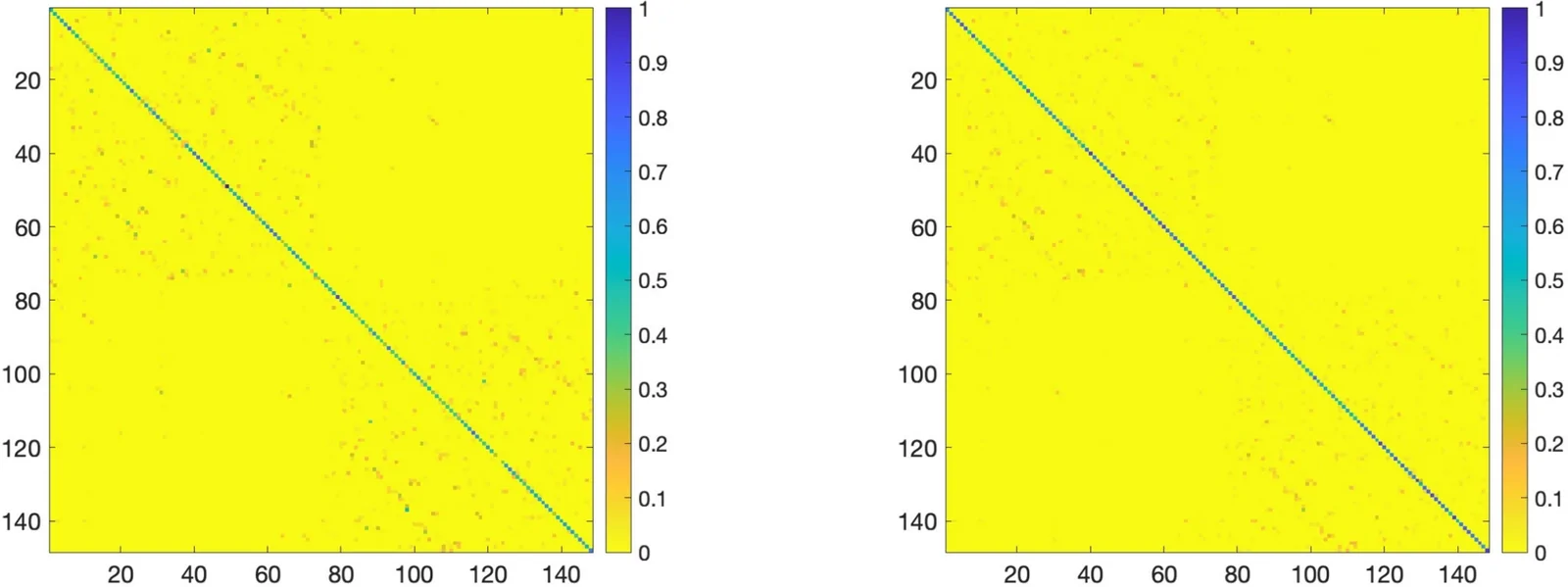
Heatmaps of the confusion matrices after the first phase using reduced dictionaries and winner-takes-all classification principle (left) and second phase based on the deflated model (right)

We compute the column-scaled matrices ${\mathsf P}^1$ and ${\mathsf P}^2$ corresponding to Phase I and Phase II, respectively, and from their heatmaps, shown in Fig. 3. It can be seen that, overall, the misclassification error is smaller at the end of Phase II than at the end of Phase I. Additional information about the misclassification is provided by the other impurity measures introduced in Sect. 4.

**Fig. 4:**
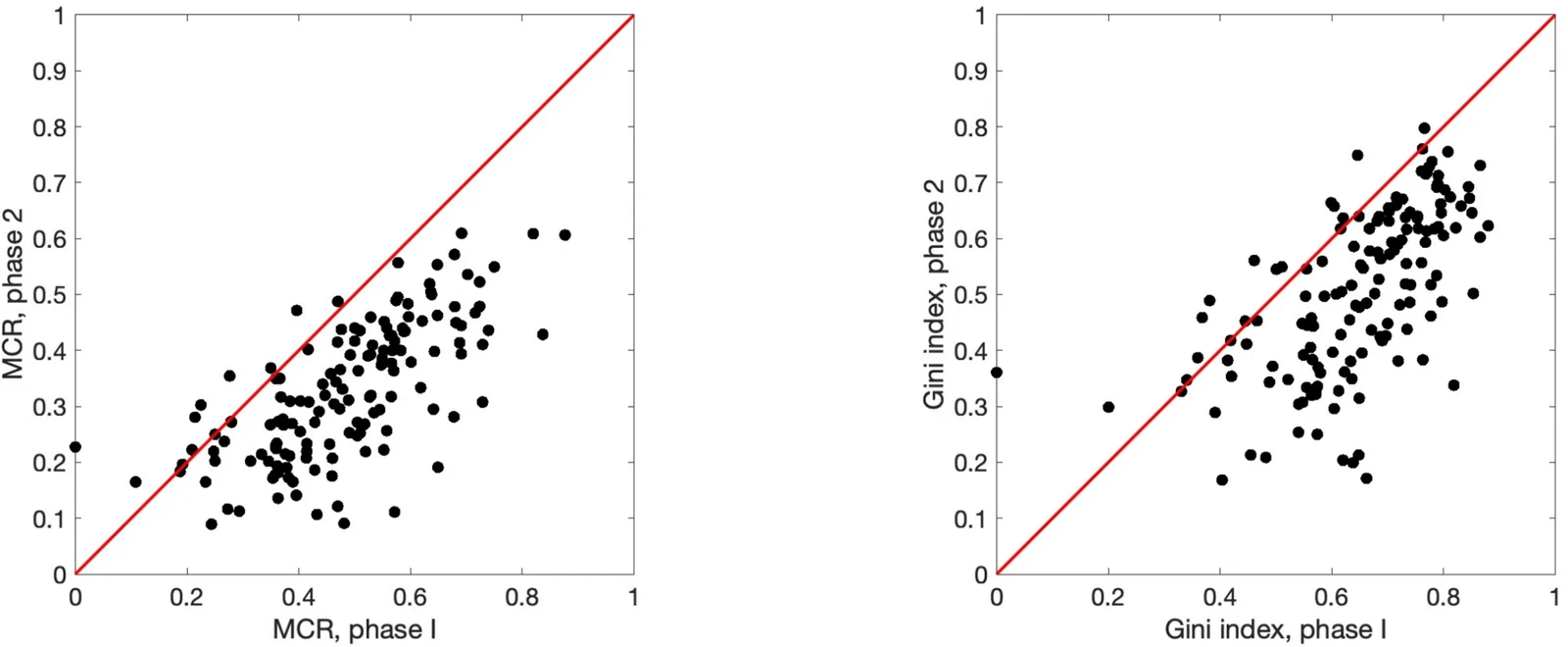
Scatter plots of the misclassification errors of each brain region (left) and the Gini indices (right). The horizontal axes correspond to the classification based on the reduced dictionaries, and the vertical plot to the deflated dictionaries. Observe that a decrease of the impurity in Phase II is indicated by the marker for brain region to be below the red diagonal line

Figure 4 shows the misclassification rates $MCR_j$ and Gini indices $G_j$ for each brain region in scatter plot form, the horizontal axis corresponding to the result at the end of Phase I, and the vertical axis corresponding to the result at the end of Phase II. Dots below the main diagonal indicate that for the corresponding brain regions the classification improves in Phase II, as we would expect and as is indeed the case for most of the brain regions, although in some rare cases the impurity is higher at the end of Phase II than at the end of Phase I.

Following the terminology used in binary classification, we define the recalls of each brain region as

63 $$\begin{aligned} s_j = 1 - MCR_j. \end{aligned}$$

**Fig. 5:**
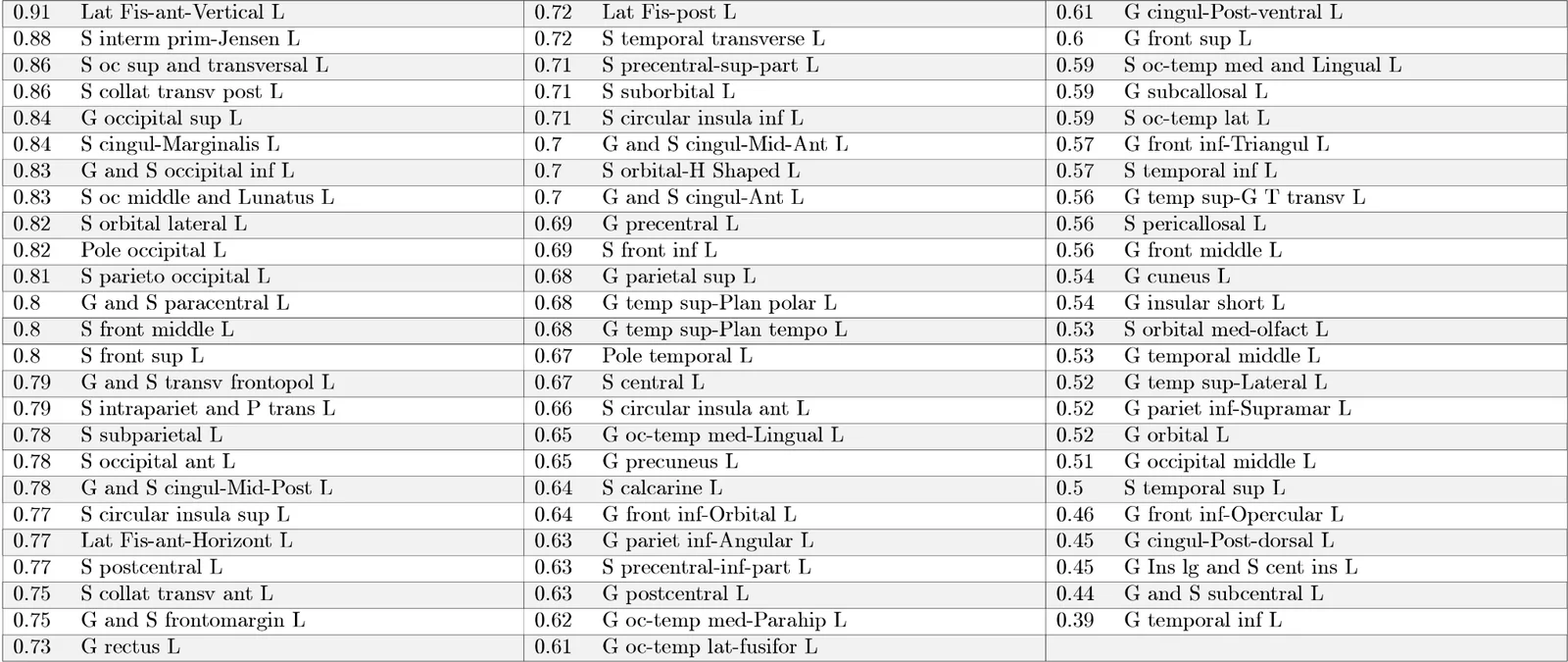
The recalls $s_j = 1-MCR_j$ of the brain regions of the left hemisphere in decreasing order. Here, both phases of the classifier algorithm were applied

In Fig. 5, the 74 Destrieux regions in the left hemisphere are listed in decreasing order of recall computed at the end of Phase II.

To gain some understanding on why some brain regions may be prone to be misclassified and to shed some light on which brain regions the confusion entails, we investigate in more depth four regions with different classification success rate, and visualize the corresponding column vectors of the matrix ${\mathsf P}= {\mathsf P}^2$, recalling that the *i*th entry of the column can be viewed as the probability of the activity in region *i* to be classified as activity in the region defining the column. If the classifier is reliable, we would expect the support of the column to be concentrated in the *i*th entry.

**Fig. 6:**
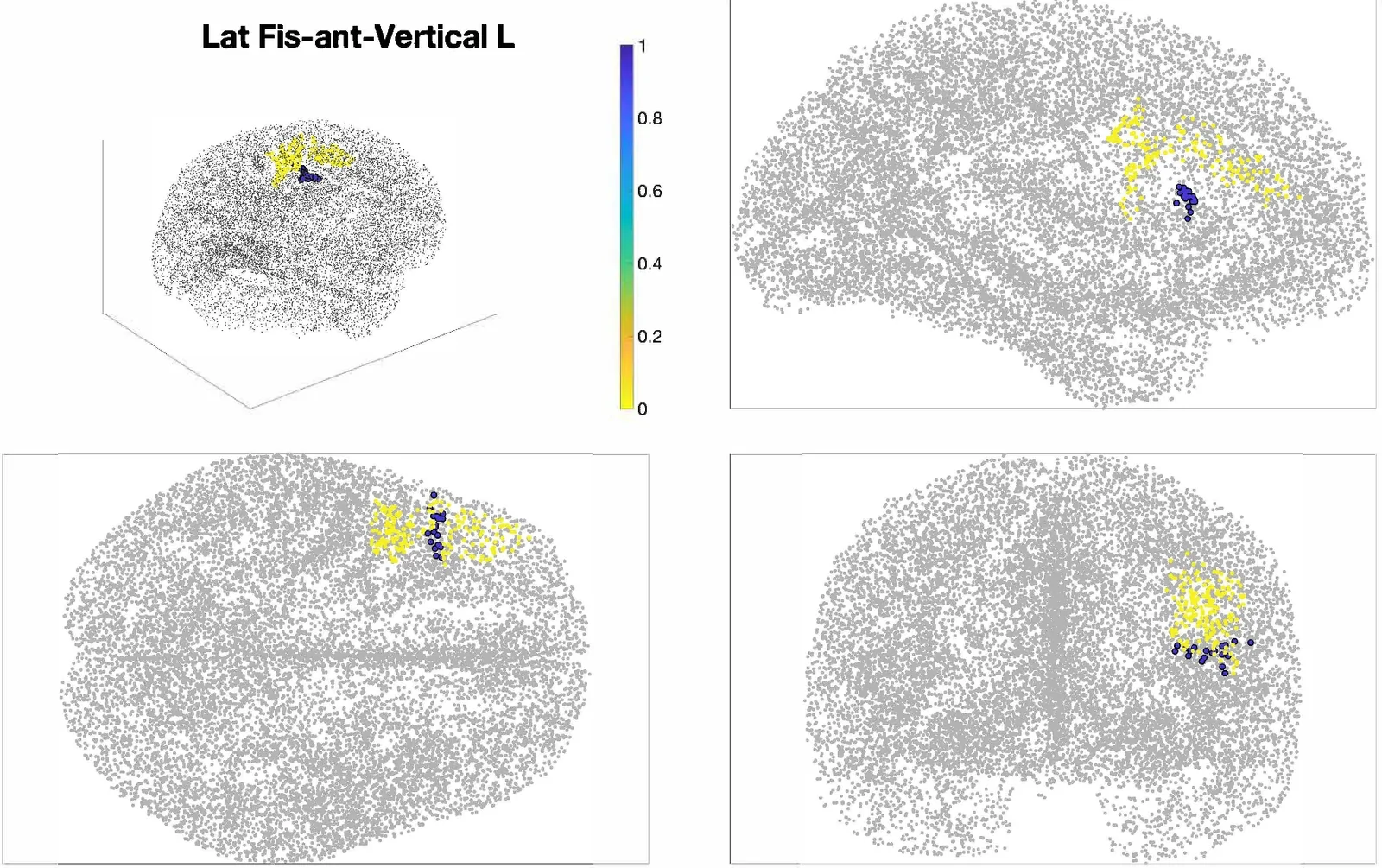
Visualization of entries $({\mathsf P}_{ij})_{i=1}^N$, of the *j*th vector, interpreted as probabilities, corresponding to vertical ramus of the anterior segment of the lateral fissure, the brain region with the highest recall value of 0.91. In this plot, as well as in Figs. 7, 8, 9, the dipole locations in the entire brain region are colored with the color corresponding to the probability of that region. In all cases, the most probable brain region (darkest shade of blue in the plot) coincides with the correct region. As expected, the confounding regions (yellow) are geometrically adjacent to the correct one

**Fig. 7:**
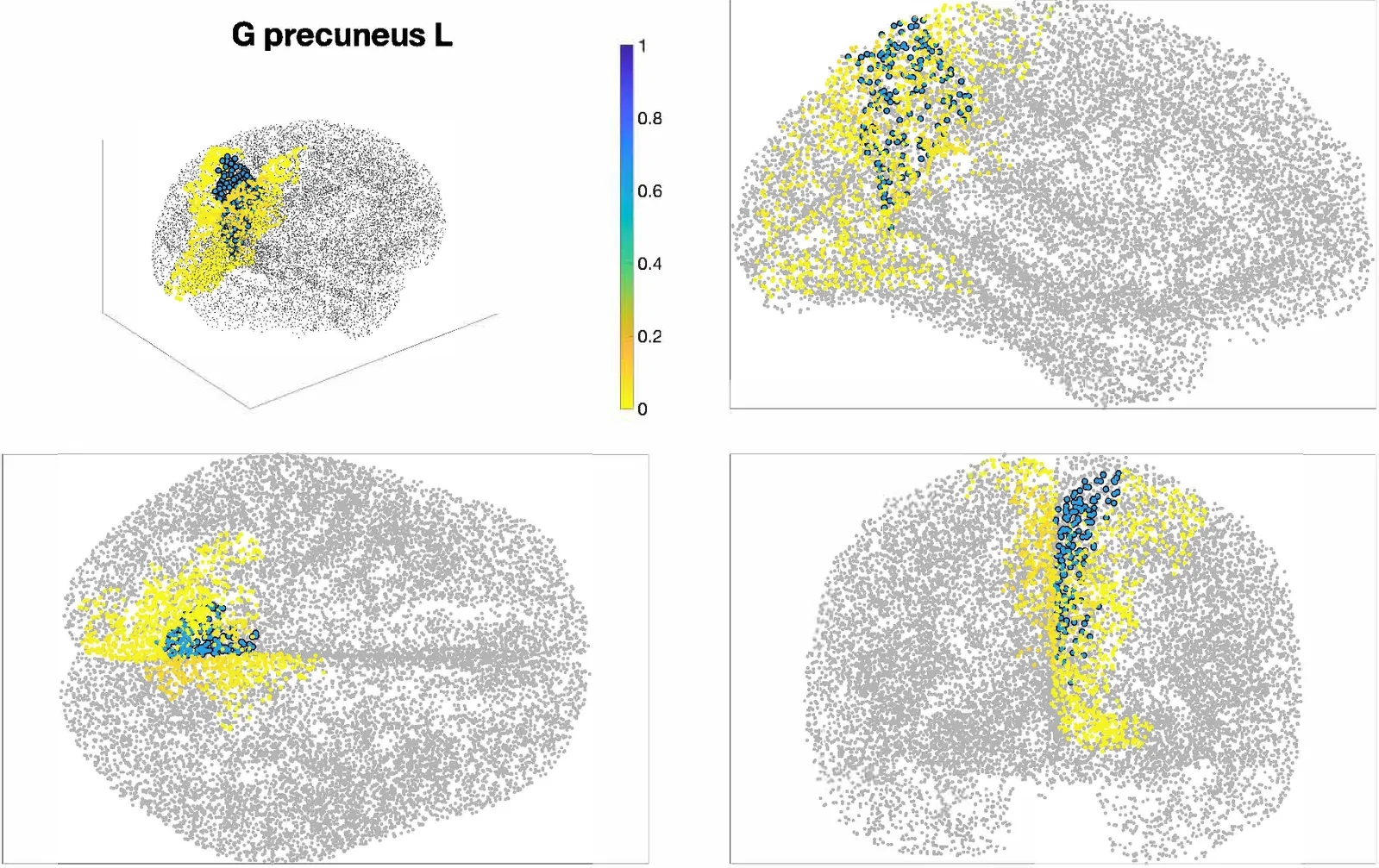
Visualization of the probabilities of the regions to be identified as left precuneus, with recall value 0.65. Observe that due to the proximity of the left precuneus to the longitudinal fissure separating the hemispheres, the misclassification includes some brain regions on the right hemisphere

**Fig. 8:**
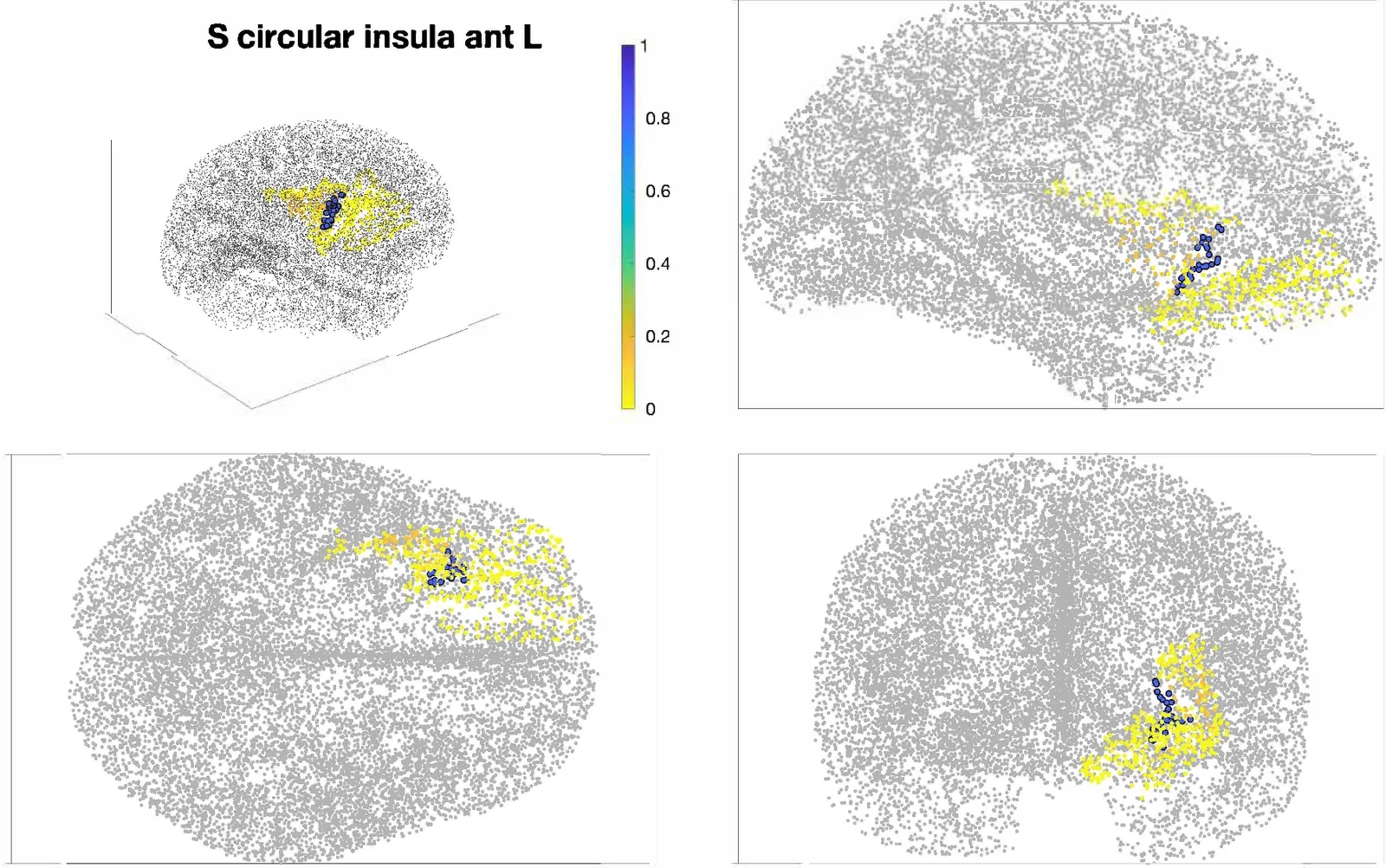
Visualization of the probabilities of brain regions to be identified as the anterior sulcus of the left circular insula. This deep brain region has the recall value 0.66

**Fig. 9:**
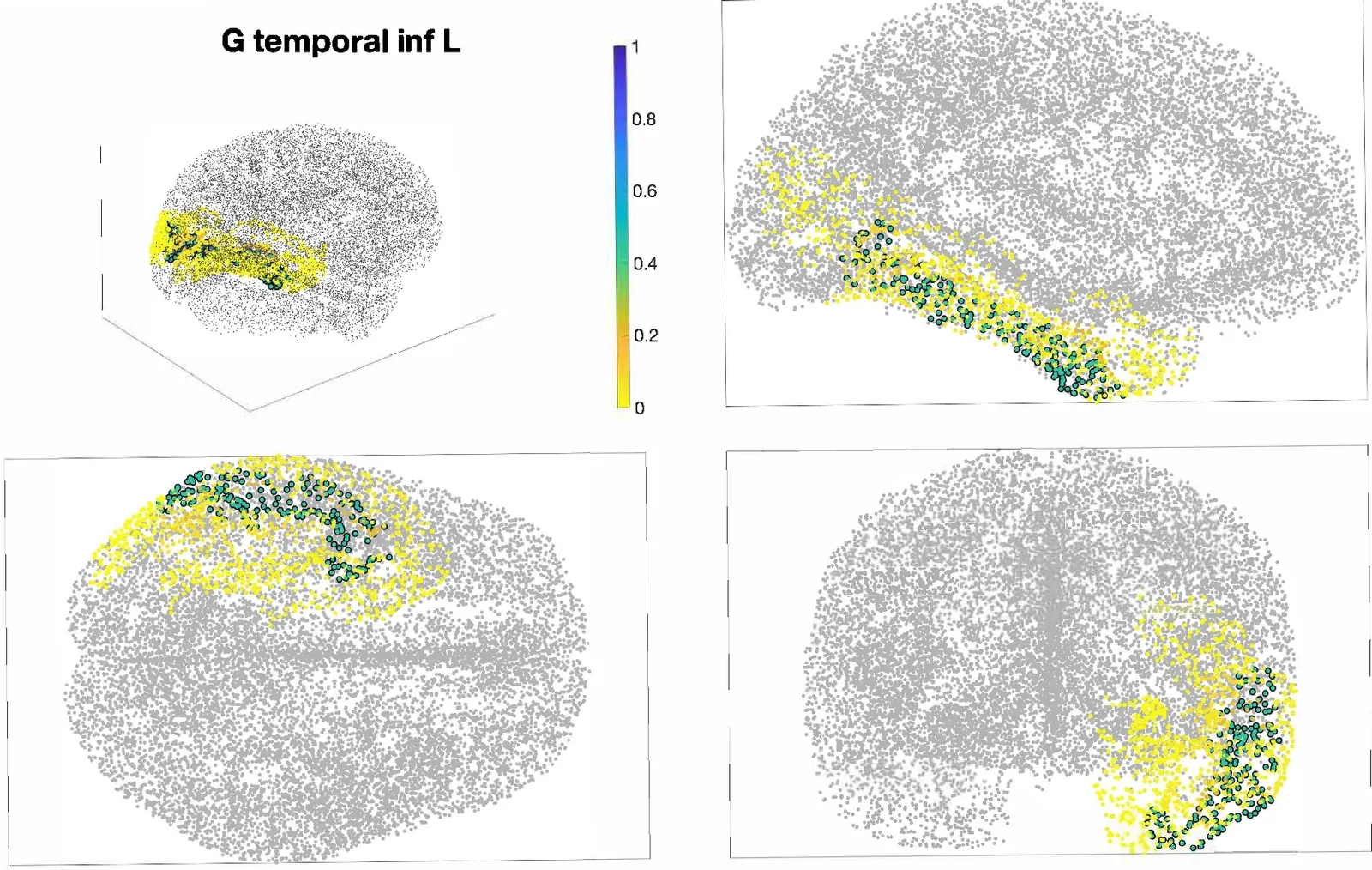
Visualization of the probabilities of brain regions to be identified as the left inferior temporal gyrus, the region with the lowest recall, 0.39, in the left hemisphere. The confusion may be due to the relatively large size of the region, as well as its position in the brain making the MEG data less sensitive to signals arising from it

Figure 6 refers to the anterior segment of the lateral fissure in the left hemisphere, the brain region having highest recall. Figures 7 and 8 correspond to the left precuneus and to the anterior sulcus of the left circular insula, two brain regions with mid-range recall values, and Fig. 9 to the inferior temporal gyrus, the brain region in the left hemisphere with the lowest recall. One reason for the low recall for the inferior temporal gyrus is its distance from the MEG sensors, contributing to low sensitivity of the MEG data to signals coming from this region. Moreover, the relatively large extension of this brain region contributes to a wide variability in the induced magnetic field, increasing the probability that the signal is mistakenly identified as coming from one of the numerous neighboring regions. The regions with the highest recalls, on the other hand, tend to be more superficial, close to the MEG the sensors, and relatively small. While most of the confusion is confined to the hemisphere where the identified region lies, we see in Fig. 7 that the proximity to the longitudinal fissure may spread the confusion across the hemispheres.

We illustrate the tree interpretation for four ROIs in the left hemisphere from the Destrieux atlas, the *middle and posterior cingulate region (sulcus and gyrus)*, the *frontal superior gyrus*, the *inferior parietal angular gyrus* and the *precuneus gyrus*. The selected brain regions play a central role in the default mode network (DMN), a focus of interest in the brain connectivity studies (Raichle 2015; Menon 2023). We point out that our analysis does not aim at suggesting any network structure between the brain regions studied here. Rather, the selection of the target regions is based on their centrality in numerous studies, and our analysis points at possible pitfalls in activity identification.

**Fig. 10:**
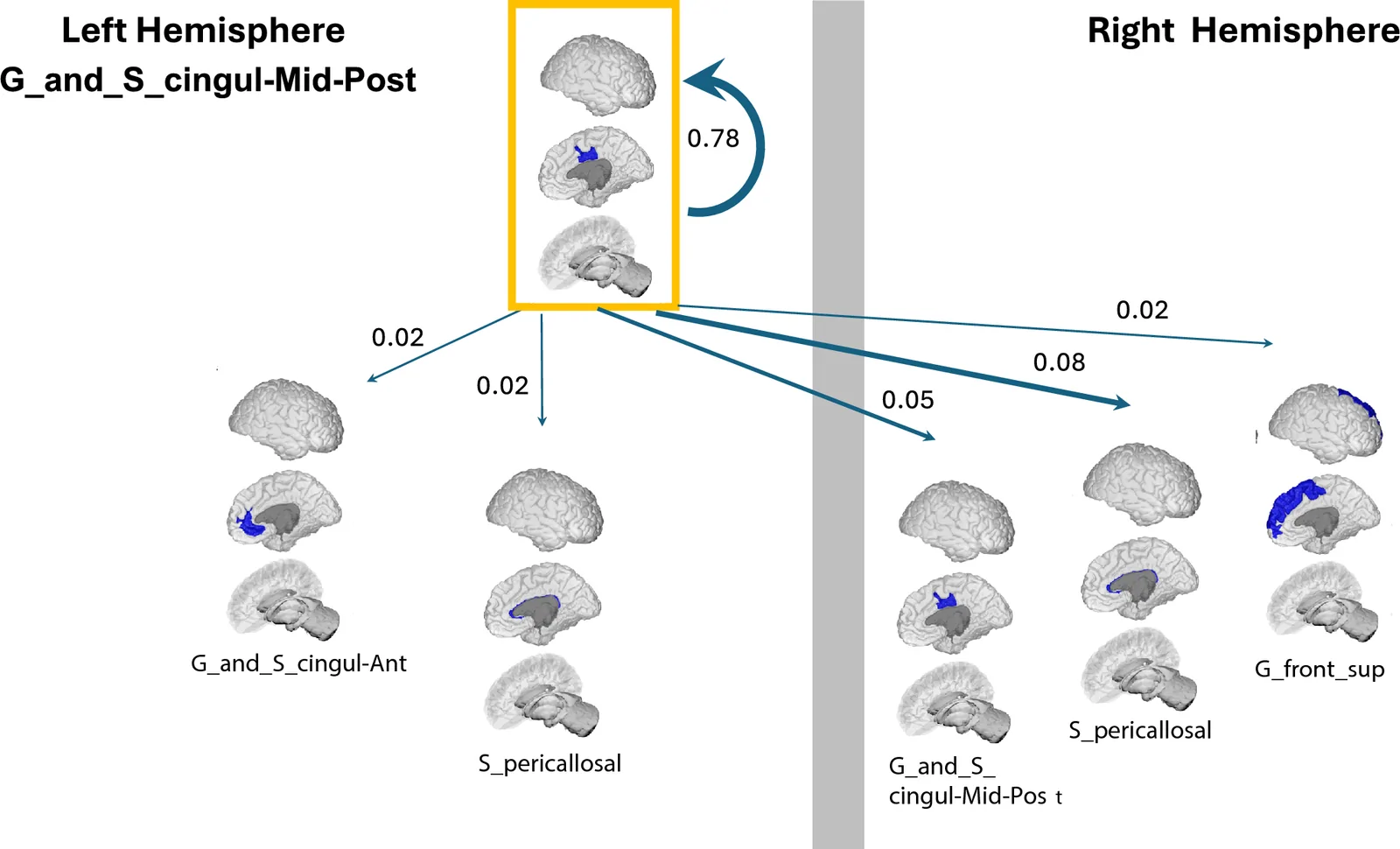
Graph representation of the attribution of activity restricted to the left mid-posterior cingulate region from MEG data. Most of the time the activated region is classified correctly. The location of the region, in the middle of the brain, close to the longitudinal fissure explains the (low probability) misattribution of the activation to nearby region in the right hemisphere

The left middle and posterior cingulate cortex and sulcus (MPCCS) play a role in some important higher-level functions, including allocation of attention, anticipation of rewards, decision-making, control of impulses, and emotions. The weighted tree associated with the left MPCCS region, shown in Fig. 10, suggests that cerebral activity localized to this brain region is very likely to be correctly and unequivocally identified from MEG measurement, as it was the case in 78% of the test cases. The anatomical location of this region, right above the pericallosal sulcus may suggest a challenge in the correct distinction between the two areas, however in our simulations only 10% of the times activity from the left MPCCS region was localized in the left or right pericallosal sulcus. The closeness of the MPCCS to the to midsagittal plane explains why 5% of the cases, the activity was attributed to the specular, and very near right mid-posterior cingulate region, which in our simulations occurred in only 5% of the cases.

**Fig. 11:**
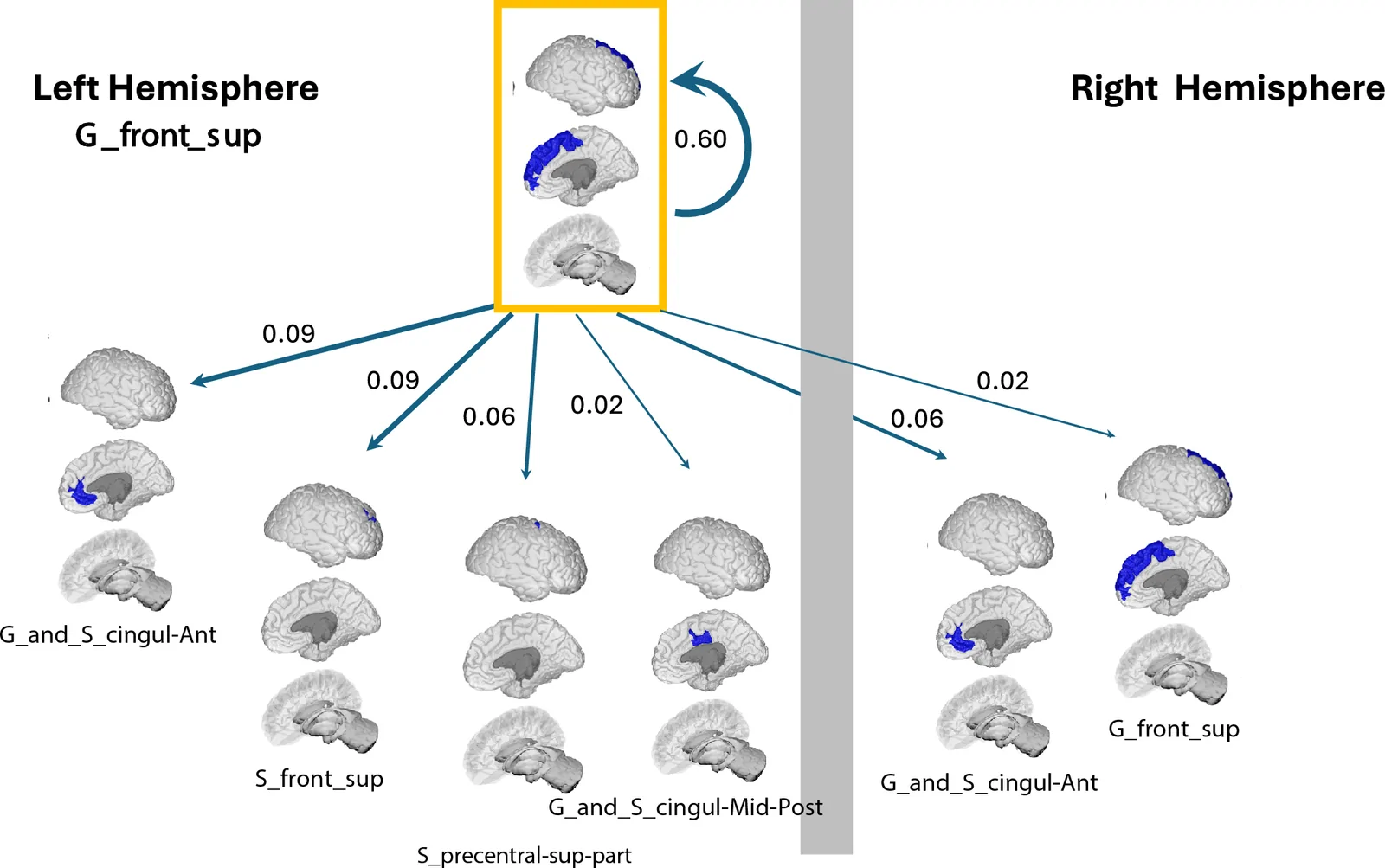
Graph representation of the attribution of activity originating inside the left frontal superior gyrus from MEG data

The left superior frontal gyrus (SFG), which is the top part of the prefrontal cortex, is a node of the cognitive control network and the default mode network. It has been hypothesized that the SFG, which is primarily associated with Brodmann areas 6, 8, and 9, plays a role in higher cognitive functions, for example working memory, self-monitoring, planning, and organization. The weighted tree associated with the left SFG region, shown in Fig. 11, indicates that the most likely regions to be confused with this ROI are, not surprisingly, the left superior frontal sulcus, the left and right cingulate anterior sulcus and gyrus, and the left precentral superior parietal sulcus, which are all anatomically very close.

The left angular gyrus (LAG) is a horseshoe shaped region located in the inferior portion of the parietal lobule, involved with involved in various cognitive functions, including language processing, spatial cognition, and memory retrieval. The identification of activity in this region from MEG signal may be attributed to the superior temporal, intraparietal and Jensen sulci, which are anatomically very close and very small, and, with a lower probability, to other regions within the parietal lobe.

The left precuneus gyrus (LPG) is located in the portion of the superior parietal lobule on the medial surface, in front of the upper portion of the occipital lobe. LPG is bounded by the parieto-occipital sulcus and by the subparietal sulcus, thus it is not surprising that these are the more frequent confounding regions in source reconstruction from MEG.

To summarize the findings, we note that the Destrieux parcellation with its distinction between sulci and gyri, based on widely accepted anatomical conventions, has been designed for the automatically labeling the whole cerebral cortex, but may be suboptimal for assessing the resolution power of MEG data for source identification.

**Fig. 12:**
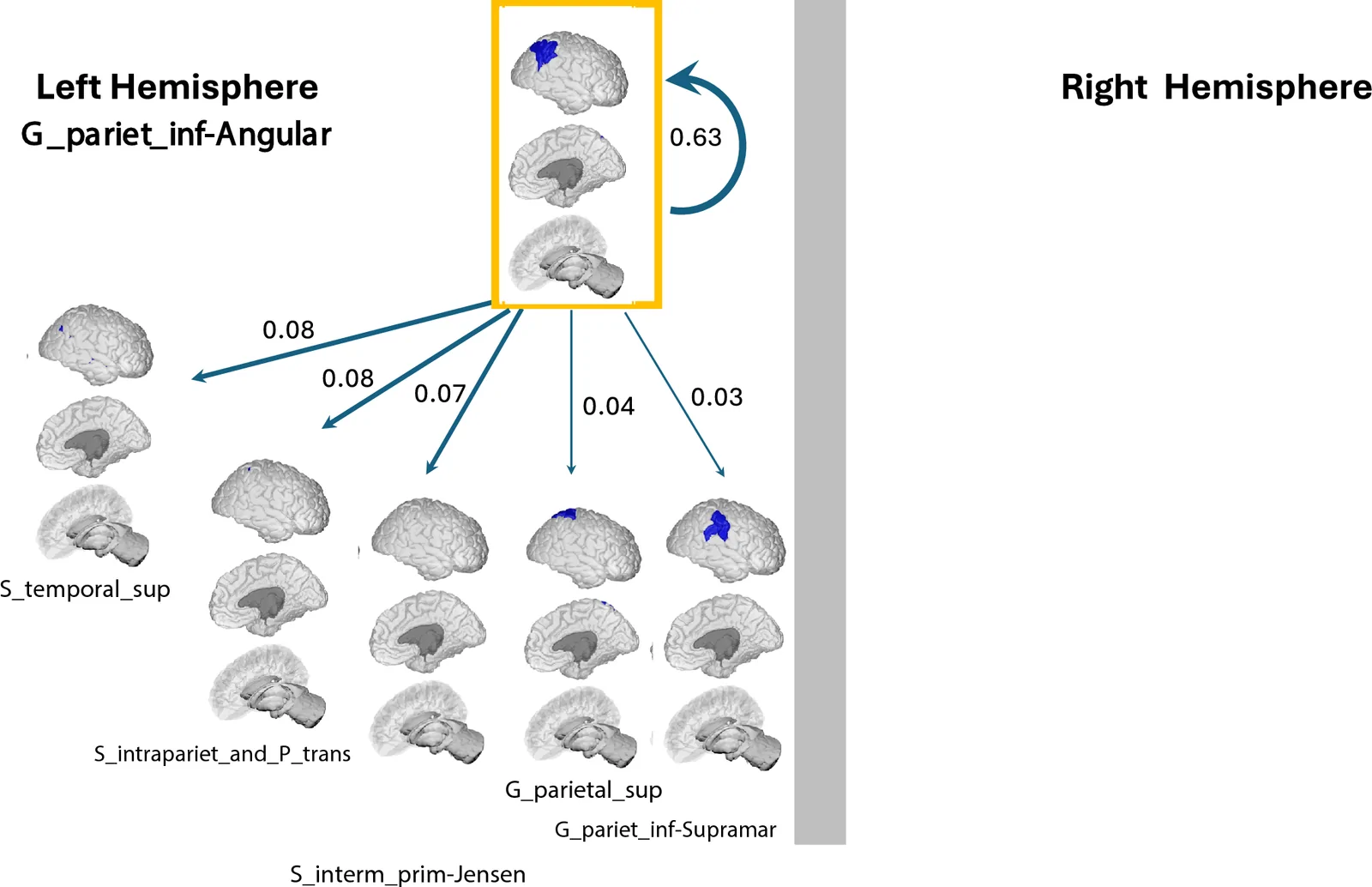
Graph representation of the attribution of activity originating inside the left angular gyrus in the parietal lobe from MEG data. This region is located away from the longitudinal fissure separating the left and right hemisphere, and consequently, no hemispheral confusion occurs

**Fig. 13:**
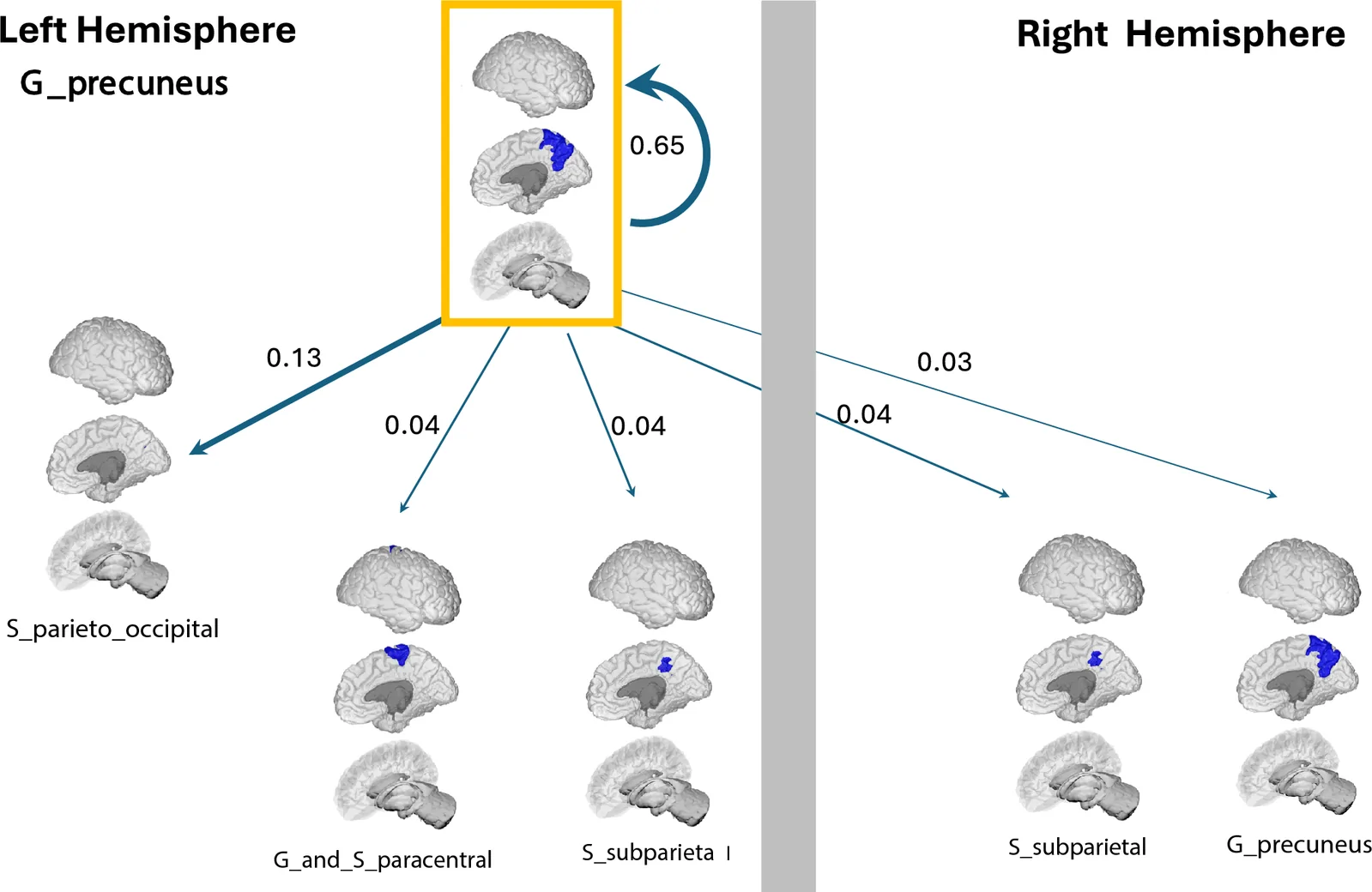
Graph representation of the attribution of activity originating inside the left precuneus gyrus from MEG data. The proximity of the left precuneus to the longitudinal fissure explain why is some case the activity is misclassified as originating in the right precuneus

## Discussion

In this article, a version of the Bayesian dictionary learning algorithm was adapted for the MEG source identification problem. The identification of each region of the the Destrieux atlas from MEG data was systematically tested through numerical simulations by generating a random patch activity in a single brain region at the time. The performance of the classifier was assessed over a suite of simulated data arising from 100 randomly activated patches for each brain region. From the point of view of the practical applications, the question was posed as an a posteriori estimate: What are the probabilities of various regions to be active, given that the winner-takes-all algorithm identifies one region? While in this question it is implicitly assumed that the activity is confined to one single region, the analysis sheds light on the success of identifying more than one activity region from the data: The testing method was posed only for having an easily quantifiable output, but it is not an inherent feature of the algorithm itself, as the algorithm does not require the winner-takes-all identification. Indeed, the output of the algorithm can be summarized in terms of the estimated variance vectors ${\boldsymbol{\theta }}$, and by thresholding several brain regions can be interpreted to be simultaneously active.

The Bayesian formulation underlying the dictionary learning algorithm opens possibilities for uncertainty quantification beyond the dictionary identification of the active region. As pointed out in Bocchinfuso et al. (2024), the first phase of the algorithm selects a few brain regions and while usually the activated one is among them, it is not necessarily the one with the highest variance value, thus the winner-take-all criterion would lead to misclassification, which is the main motivation for the addition of Phase II. Since it may be argued that the solution at the end of Phase I is a maximum a posteriori (MAP) estimate, which may be seen as not a true Bayesian solution because of its dependence on the model parametrization, an alternative approach is to find instead the computationally more expensive sampling-based posterior mean estimate. In Calvetti and Somersalo (2025), this question was addressed by means of developing a fast MCMC sampler suitable for problems with priors favoring group sparsity, and it was shown that the current model for MEG is amenable for this approach. While the sampling based method may not be as suitable for processing large time series data as the current dictionary learning method, it provides a way to assess the reliability of the algorithm at selected control points.

As indicated in the discussion above, this work opens a way for several improvements and extensions, as well as for further investigations. In the present work, the dipole directions were fixed to correspond to the organization of cortical neurons preferably normal to the cortical surface, an assumption that is not uncommon in the literature, see, e.g., Giri et al. (2025). While often a reasonable assumption, a more general approach is to implement the normal direction as a preferred but not enforced direction, e.g., using anatomical prior models (Calvetti et al. 2015, 2019). Incorporating such priors has been done in the source localization framework in the cited articles, however in the dictionary learning approach it is not immediate and requires more work. Another extension of the present work is to identify simultaneously several active regions. While the algorithm proposed here does not require the use of the winner-takes-all type classification, selecting judiciously the number of active brain regions is an open question beyond the current paper, and would require extensive and well-organized simulation studies. Likewise, analyzing thoroughly and reliably the sensitivity of the classification performance of the algorithm with different SNR levels that may depend on the source position requires a significant amount of simulations to avoid misinterpretations due to the randomization of the source models. Finally, the results in this paper suggest that the anatomical atlas such as Destrieux atlas may not be a natural one for ROI identification from MEG data, in particular because nearby regions may be almost indistinguishable by the data, while at the same time they may be functionally similar. As a conclusion, the selected parcellation should be adjusted according to the goals of the mapping task. A major motivation for this work is to find a relatively fast algorithm for brain region identification in the time-dependent setting where new data arrives at a high pace. Here, the analysis was restricted to a single time slice, but incorporating the temporal direction is one of the possible future challenges.

## Data Availability

No datasets were generated or analysed during the current study.

## Appendix

In this appendix, we give a brief overview of the IAS algorithm. Consider a linear inverse problem with additive Gaussian noise,

64 $$\begin{aligned} {\boldsymbol{b}}= {\mathsf A}{\boldsymbol{x}}+ {\boldsymbol{e}}\end{aligned}$$

where ${\mathsf A}\in {\mathbb {R}}^{m\times n}$ is given and ${\boldsymbol{e}}$ is a realization of a random noise variable ${\boldsymbol{E}}\sim {\mathcal {N}}(0,\sigma ^2 {\mathsf I}_n)$. The goal is to find an estimate ${\boldsymbol{x}}\in {\mathbb {R}}^n$ that is sparse, or more generally, compressible, i.e., most of the components of ${\boldsymbol{x}}$ are zero or at least below a small threshold. The IAS algorithm is based on a conditionally Gaussian prior model,assuming that ${\boldsymbol{x}}$ is a realization of a random variable ${\boldsymbol{X}}$,

65 $$\begin{aligned} {\boldsymbol{X}}\sim {\mathcal {N}}(0,{\mathsf D}_{\boldsymbol{\theta }}), \end{aligned}$$

where ${\mathsf D}_{\boldsymbol{\theta }}$ is a diagonal matrix with diagonal entries ${\mathsf D}_{jj} = \theta _j>0$, and the parameter vector ${\boldsymbol{\theta }}$ itself is a realization of a random vector ${\boldsymbol{\Theta }}\in {\mathbb {R}}^n$ with mutually independent components characterized by a heavy tailed probability distribution, such as the Generalized Gamma distribution,

66 $$\begin{aligned} \Theta _j \sim \theta _j^{r\beta -1}\textrm{exp}\left( -\left( \frac{\theta _j}{\vartheta _j}\right) ^r\right) , \quad r\ne 0, \end{aligned}$$

where, unlike in formula (38), we assume the shape parameter *β* to be the same for every *j*. These assumptions give rise to the joint prior probability density of the pair $({\boldsymbol{X}},{\boldsymbol{\Theta }})$,

67 $$\begin{aligned}&\pi _{{\boldsymbol{X}},{\boldsymbol{\Theta }}}({\boldsymbol{x}},{\boldsymbol{\theta }}) = \pi _{{\boldsymbol{X}}\mid {\boldsymbol{\Theta }}}({\boldsymbol{x}}\mid {\boldsymbol{\theta }})\pi _{\boldsymbol{\Theta }}({\boldsymbol{\theta }}) \nonumber \\&\propto \textrm{exp}\left( -\frac{1}{2}\sum _{j=1}^n\frac{x_j^2}{\theta _j} -\sum _{j=1}^n\left( \frac{\theta _j}{\vartheta _j}\right) ^r \right. \nonumber \\&\left. - \left( \frac{3}{2} - r\beta \right) \sum _{j=1}^n\log \theta _j \right) . \end{aligned}$$

The philosophy behind the choice of hypermodel is that the components $\Theta _j$ are positive, have a small expected value, yet the heavy tail admits significantly large but rare outlier values. This in line with the prior belief that most of the variances of the components $X_j$ should be small, while it should be possible for a few of them to have large variance. In the Bayesian framework, we let the data decide which outlier values best explain the data.

Building the likelihood on the observation model (64) with the assumption that ${\boldsymbol{X}}$ and the noise ${\boldsymbol{E}}$ are mutually independent, we have

68 $$\begin{aligned} \pi _{{\boldsymbol{B}}\mid {\boldsymbol{X}}}({\boldsymbol{b}}\mid {\boldsymbol{x}}) \propto \textrm{exp}\left( -\frac{1}{2\sigma ^2}\Vert {\boldsymbol{b}}- {\mathsf A}{\boldsymbol{x}}\Vert ^2 \right) , \end{aligned}$$

and by Bayes’ theorem asserting that the posterior density of the pair $({\boldsymbol{B}},{\boldsymbol{\Theta }})$ is proportional to the product of the prior (67) and the likelihood (68), we may write

69 $$\begin{aligned}&\pi _{{\boldsymbol{X}},{\boldsymbol{\Theta }}\mid {\boldsymbol{B}}}({\boldsymbol{x}},{\boldsymbol{\theta }}\mid {\boldsymbol{b}}) \nonumber \\& \propto\textrm{exp}\left( -\frac{1}{2\sigma ^2}\Vert {\boldsymbol{b}}- {\mathsf A}{\boldsymbol{x}}\Vert ^2-\frac{1}{2}\sum _{j=1}^n\frac{x_j^2}{\theta _j} -\sum _{j=1}^n\left( \frac{\theta _j}{\vartheta _j}\right) ^r \right. \nonumber \\&\left. - \left( \frac{3}{2} - r\beta \right) \sum _{j=1}^n\log \theta _j\right) . \end{aligned}$$

A Maximum A Posteriori (MAP) estimate $({\boldsymbol{x}}_\textrm{MAP},{\boldsymbol{\theta }}_\textrm{MAP})$ maximizes the above expression, or, equivalently, the minimizer of the functional

70 $$\begin{aligned}&{\mathscr {E}}({\boldsymbol{x}},{\boldsymbol{\theta }}) = \frac{1}{2\sigma ^2}\Vert {\boldsymbol{b}}- {\mathsf A}{\boldsymbol{x}}\Vert ^2+\frac{1}{2}\sum _{j=1}^n\frac{x_j^2}{\theta _j} \nonumber \\&+\sum _{j=1}^n\left( \frac{\theta _j}{\vartheta _j}\right) ^r + \left( \frac{3}{2} - r\beta \right) \sum _{j=1}^n\log \theta _j \end{aligned}$$

The IAS algorithm is an alternating direction optimization algorithm, consisting of alternating the following steps: Starting from an initial value ${\boldsymbol{\theta }}^0={\boldsymbol{\vartheta }}$, and setting the counter to *k=0*,

1. Update ${\boldsymbol{x}}={\boldsymbol{x}}_{k+1}$ by solving the least squares problem
  71 $$\begin{aligned} {\boldsymbol{x}}_{k+1} = \textrm{argmin}\left\{ \frac{1}{2\sigma ^2}\Vert {\boldsymbol{b}}- {\mathsf A}{\boldsymbol{x}}\Vert ^2+\frac{1}{2}\sum _{j=1}^n\frac{x_j^2}{\theta _j} \right\} ,\quad {\boldsymbol{\theta }}={\boldsymbol{\theta }}_k; \end{aligned}$$
2. Update $\theta ={\boldsymbol{\theta }}_{k+1}$ by solving the first order optimality condition in a component-wise fashion
  72 $$\begin{aligned} \frac{\partial {\mathcal {E}}}{\partial \theta _j} = -\frac{x_j^2}{2\theta _j^2} +r\frac{\theta _j^{r-1}}{\vartheta _j^r}+\left( \frac{3}{2} - r\beta \right) \frac{1}{\theta _j} = 0, \quad {\boldsymbol{x}}={\boldsymbol{x}}_{k+1}. \end{aligned}$$

The properties of the IAS algorithm are well studied (Calvetti and Somersalo 2023); below we summarize the most relevant features for the current application.

For *r=1*, the IAS algorithm to converge to the unique minimizer of the strictly convex objective function, while for *r<1*, multiple local minima may occur, but the convergence is guaranteed with known convergence rates (Calvetti et al. 2020b). A hybrid version of the algorithm to avoid early stopping at local minima has been proposed in Calvetti et al. (2020a). For *r=1*, the parameter $\eta = \beta -3/2>0$ controls the sparsity of the solution, and in the limit as $\eta \rightarrow 0^+$, the solution converges to a weighted version of the classical $\ell ^1$-penalized solution (Calvetti et al. 2019). The role of the hyperpriors $\vartheta _j$ is related to the sensitivity scaling. It was shown in Calvetti et al. (2019) that under a relatively general assumptions, the parameters $\vartheta _j$ should be chosen proportional to the sensitivity of the forward map ${\boldsymbol{x}}\mapsto {\mathsf A}{\boldsymbol{x}}$ to the *j*th component, which in the linear model corresponds to the square of the norm of the *j*th column of the matrix ${\mathsf A}$. In particular, assuming that the columns of the matrix ${\mathsf A}$ are scaled to unity as in our model, the hyperparameters $\vartheta _j$ can be set to a constant value. A more detailed discussion can be found in the cited references.
